# Multicomponent Intervention for Overactive Bladder in Women: A Randomized Clinical Trial

**DOI:** 10.1001/jamanetworkopen.2024.1784

**Published:** 2024-03-13

**Authors:** Satoshi Funada, Yan Luo, Ryuji Uozumi, Norio Watanabe, Takayuki Goto, Hiromitsu Negoro, Kentaro Ueno, Kentaro Ichioka, Takehiko Segawa, Tatsuo Akechi, Osamu Ogawa, Shusuke Akamatsu, Takashi Kobayashi, Toshi A. Furukawa

**Affiliations:** 1 Department of Urology, Kyoto University Graduate School of Medicine, Kyoto, Japan; 2 Department of Health Promotion and Human Behavior, School of Public Health, Graduate School of Medicine, Kyoto University, Kyoto, Japan; 3 Department of Psychiatry and Cognitive-Behavioral Medicine, Nagoya City University Graduate School of Medical Sciences, Aichi, Japan; 4 Department of Industrial Engineering and Economics, Tokyo Institute of Technology, Tokyo, Japan; 5 Department of Psychiatry, Soseikai General Hospital, Kyoto, Japan; 6 Department of Urology, Institute of Medicine, University of Tsukuba, Ibaraki, Japan; 7 Department of Biomedical Statistics and Bioinformatics, Kyoto University Graduate School of Medicine, Kyoto, Japan; 8 Ichioka Urological Clinic, Kyoto, Japan; 9 Department of Urology, Kyoto City Hospital, Kyoto, Japan

## Abstract

**Importance:**

Although the cognitive components of behavioral therapy for overactive bladder (OAB) are widely recognized, there is a lack of studies evaluating the effectiveness of multicomponent interventions that include cognitive components as a treatment for OAB.

**Objective:**

To examine the efficacy of a multicomponent intervention in improving health-related quality of life (HRQOL) for women with moderate to severe OAB.

**Design, Setting, and Participants:**

This multicenter, open-label, randomized clinical trial was conducted in Japan among women aged 20 to 80 years who had moderate to severe OAB. Participants were recruited from 4 institutions between January 16, 2020, and December 31, 2022, through self-referral via advertisement or referral from the participating institutions.

**Interventions:**

Participants were randomized 1:1 by minimization algorithm using an internet-based central cloud system to four 30-minute weekly sessions of a multicomponent intervention or waiting list. Both groups continued to receive baseline treatment throughout the study period.

**Main Outcomes and Measures:**

The primary outcome was the least-squares mean changes from baseline through week 13 in HRQOL total scores of the OAB questionnaire between 2 groups. Secondary outcomes included OAB symptom score and frequency volume chart.

**Results:**

A total of 79 women were randomized to either the intervention group (39 participants; mean [SD] age, 63.5 [14.6] years) or the waiting list control group (40 participants; mean [SD] age, 63.5 [12.9] years). One participant from each group dropped out from the allocated intervention, while 5 participants in the intervention group and 2 in the control group dropped out from the primary outcome assessment at week 13. Thirty-six participants (92.3%) in the intervention group and 35 (87.5%) in the control group had moderate OAB. The change in HRQOL total score from baseline to week 13 was 23.9 points (95% CI, 18.4-29.5 points) in the intervention group and 11.3 points (95% CI, 6.2-16.4 points) in the waiting list group, a significant difference of 12.6 points (95% CI, 6.6-18.6 points; *P* < .001). Similar superiority of the intervention was confirmed for frequency of micturition and urgency but not for OAB symptom score.

**Conclusions and Relevance:**

These findings demonstrate that a multicomponent intervention improves HRQOL for women with moderate to severe OAB and suggest that the cognitive component may be an effective treatment option for women with OAB.

**Trial Registration:**

UMIN Clinical Trials Registry Identifier: UMIN000038513

## Key Points

**Question:**

What is the efficacy of a multicomponent intervention with cognitive components in improving health-related quality of life (HRQOL) for women with moderate to severe overactive bladder (OAB)?

**Findings:**

In this randomized clinical trial of 79 women, those who underwent four 30-minute weekly sessions of a multicomponent intervention had a significantly and clinically significant improvement in HRQOL scores compared with the waiting list control group.

**Meaning:**

These findings provide evidence to support a multicomponent intervention with cognitive components as an effective treatment option for women with moderate to severe OAB by improving HRQOL.

## Teaser

This randomized clinical trial examines the efficacy of a multicomponent intervention in improving health-related quality of life for women with moderate to severe OAB.

## Introduction

Overactive bladder (OAB) is characterized by urgency of urination, with or without urgency incontinence, often accompanied by frequency and nocturia.^1^ The prevalence of OAB is estimated to range between 10% and 20% in women, with a higher incidence observed among older individuals.^2,3,4^ Overactive bladder may substantially affect a person’s life, primarily through a reduction in health-related quality of life (HRQOL).^5^ Moreover, OAB imposes a substantial economic burden, with estimated costs of OAB with urgent urinary incontinence (UUI) in the US reaching $65.9 billion in 2007, $76.2 billion in 2015, and $82.6 billion in 2020.^6^ Compounding the challenges, a considerable proportion of patients with OAB, ranging from 43% to 83%, discontinue pharmacotherapy within 1 month due to inefficacy or adverse events.^7^

As a first-line therapy for OAB, behavioral therapy encompasses lifestyle modifications, bladder training, and pelvic floor muscle training.^8,9^ A systematic review highlighted the superior efficacy and safety of behavioral therapy in treating OAB and UUI in women compared with anticholinergic medications.^10,11^ However, despite its observed effectiveness, behavioral therapy often plays only some secondary roles after drug therapy. This treatment gap may stem, in part, from the lack of structured treatment manuals guiding the appropriate selection and implementation of behavioral therapy for OAB management and the resultant perceived cumbersomeness or unfamiliarity with this type of therapy on the part of clinicians.

To overcome this discrepancy, we have focused on the potential of cognitive behavior therapy (CBT) as a treatment for OAB. Originally developed in the 1960s to treat depression, CBT comprises structured sessions that foster collaborative empiricism between therapists and patients.^12^ The efficacy of CBT extends beyond psychological disorders, with successful applications in functional diseases such as irritable bowel disease^13^ and chronic pain.^14^ Urinary voiding, previously considered solely a sensory-driven process, is now recognized to be influenced by cognitive processes.^15^ Given these insights, we hypothesized that modifying specific cognitive processes through a multicomponent intervention may hold promise as an effective treatment approach for OAB. In previous work by our study group, we developed a multicomponent intervention with cognitive components specifically tailored for drug-resistant OAB and evaluated its clinical feasibility and acceptability.^16^ Although 2 systematic reviews focused on the cognitive components of behavioral therapy for OAB, the included studies in the reviews did not actually perform cognitive intervention.^17,18^

To establish an innovative program for OAB treatment, we integrated CBT techniques and developed a structured treatment manual based on conventional behavioral therapy. We hypothesized that our multicomponent intervention with cognitive components would show greater improvement in OAB disease-specific HRQOL than a waiting list control. The present study was limited to women because the pathophysiology differs between men and women. The aim of this randomized clinical trial (RCT) was to evaluate the efficacy of our program in treating women with moderate to severe OAB.

## Methods

### Study Design

This study was a randomized, controlled, open-label, multicenter, parallel-group superiority trial conducted at Kyoto University Hospital, Kyoto City Hospital, Kyoto Min-Iren Asukai Hospital, and Ichioka Urological Clinic. The study protocol (Supplement 1) was approved by the ethics committee of Kyoto University Graduate School of Medicine (No. C1423), has been registered in the clinical trial registry (UMIN000038513), and is available elsewhere.^19^ The report has adhered to the Consolidated Standards of Reporting Trials (CONSORT) reporting guideline. Written informed consent was obtained from all participants.

### Participants

Participants were recruited through self-referral via advertisements or referral at the aforementioned institutions between January 16, 2020, and December 31, 2022. Inclusion criteria were as follows: (1) a woman aged 20 to 80 years; (2) diagnosis of OAB based on total OAB symptom score (OABSS) of higher than 6 (indicating moderate to severe severity)^20^; (3) an Eastern Cooperative Oncology Group Performance Status of 0; and (4) ability to comprehend explanations and provide written informed consent. Participants were included regardless of their pharmacotherapy status for OAB. Exclusion criteria included (1) bladder abnormalities, (2) urinary tract infection, (3) previous surgery for urinary incontinence, (4) pregnancy, (5) inability to understand Japanese, (6) depression, (7) dementia, and (8) deemed unsuitable for participation by the researchers.

### Randomization and Masking

Following the screening process, participants were randomized 1:1 to either the multicomponent intervention group or the waiting list group using the University Hospital Medical Information Network Data and Information Center of Clinical Research cloud system,^21^ an internet-based central randomization system. A minimization algorithm was used to ensure balance across age groups (20-64 years vs 65-80 years), baseline OAB severity (moderate [OABSS of 6-11 points] vs severe [OABSS of 12-15 points]), treatment status (no treatment vs past treatment vs current treatment), types of planned intervention mode (face-to-face vs online remote session), and treating institutions. Participants and the therapist were not masked, while statisticians were masked to the group allocation during the analyses.

### Intervention and Waiting List Control Groups

For the intervention group, we developed a multicomponent therapy program consisting of 4 sessions (each lasting 30 minutes) and 6 techniques, including cognitive and behavioral skills, implemented over a 4-week period.^16,19^ We defined it as a multicomponent intervention for OAB because its defining characteristics were the included cognitive-behavioral components. The 6 techniques included (1) self-monitoring of urinary habits using frequency volume charts; (2) educational sessions on the normal urinary tract system, abnormal voiding function, the epidemiology and physiology of OAB, and a CBT model for OAB (eFigure 1 in Supplement 2); (3) lifestyle modifications such as restrictions on water and coffee intake; (4) pelvic floor muscle training; (5) exposure-based bladder training to treat OAB using a graded exposure task as the behavioral experiment to correct distorted or exaggerated beliefs about incontinence and other urination-related fears (eFigure 2 in Supplement 2); and (6) relapse prevention, involving future planning and encouragement to continue practicing the taught techniques. The protocol (Supplement 1) provides detailed information about each session, which is divided into 3 parts: review of homework, introduction of new techniques, and review of the current day’s session. All sessions were delivered individually either face-to-face at the respective institutions or via teleconferencing. The therapist, a urologist (S.F.) with 10 years of experience in urologic treatment and a Japanese board-certified instructor, followed a treatment manual developed specifically for this study to ensure intervention reproducibility. Worksheets were provided to facilitate the acquisition of each technique and completion of homework. A procedure checklist was used by the therapist during each session to ensure adherence to the treatment protocol. In the waiting list control group, participants had never received a multicomponent intervention before or during the trial and received it for the first time after the end of the trial. In both groups, participants were allowed to continue pharmacotherapy that had been initiated for more than 6 weeks before the start of this trial without changing the dose.

### Outcomes

Outcomes were assessed at baseline (week 0), immediately after the intervention (week 5), and during follow-up visits (week 9 and week 13) using self-reported questionnaires in paper form or via the REDCap system (Vanderbilt University) using a tablet. The participants used a room separate from the therapist at the time of questionnaire completion. A small incentive of a ¥1000 gift card (approximately US $7) was provided at weeks 5, 9, and 13.

The primary outcome measure was the change in HRQOL total scores assessed by the Overactive Bladder Questionnaire (OAB-q) from baseline to week 13.^22^ The OAB-q evaluates OAB-related quality-of-life outcomes through 33 questions and 6 subscales that assess symptom bother, coping behaviors, concerns or worries, sleep patterns, social interaction, and total HRQOL score. Each subscale score is transformed from 0 to 100 points, with higher scores indicating improvement, except for the symptom bother subscale, where higher scores indicate more bothersome symptoms. The minimal important change (MIC) of the OAB-q was reported to be 10 points.^23^ Secondary outcome measures included (1) other subscales of the OAB-q; (2) OABSS^20^; (3) King’s Health Questionnaire (KHQ)^24^; (4) Hospital Anxiety and Depression Scale (HADS)^25^; (5) EuroQol 5-dimensional and 5-level questionnaire^26^; (6) frequency volume chart documenting voiding frequency, urgency, and incontinence^27^; (7) Patient Global Impression-Improvement^28^; (8) Patient Global Impression-Severity^28^; (9) treatment satisfaction; (10) dropout rate; and (11) change in pharmacotherapy. Serious adverse events were reported to the principal investigator and the ethics committee by the trial physician. The protocol (Supplement 1) provides detailed information about each outcome, including the linguistic validation.

### Statistical Analysis

The sample size was calculated to detect a moderate effect size of 0.5 between the groups with a 2-sided significance level of *P* < .05, as determined using *t* test, and 80% power. Considering a 15% dropout rate, the target sample size was set at 150 participants. However, due to recruitment delays caused by the COVID-19 pandemic and the depletion of the research grant, recruitment was suspended after reaching 79 participants. All analyses were conducted based on the intention-to-treat principle, considering all participants as randomized regardless of whether they received the assigned treatment. No interim analyses were performed. For continuous outcomes, a mixed-effects model with repeated measures (MMRM) analysis was used to estimate the least-squares mean (LSM) difference in HRQOL total score changes of the OAB-q from baseline to weeks 5, 9, and 13 for between-group comparisons. This model assumed that the primary outcome data were missing at random and included study group (intervention and control), assessment points (week 5, week 9, and week 13), OAB severity (moderate and severe), baseline score, and the interaction between study group and assessment points. The primary analysis focused on the week 13 assessment. The proportion of patients showing the MIC or greater improvement was calculated using the reported MIC of the OAB-q HRQOL total scores in both the intervention and control groups.^29^ The standardized mean difference (SMD) was calculated by dividing the mean difference by the pooled SD. Conventionally, an SMD of 0.2, 0.5, and 0.8 is interpreted as representing a small, medium, and large effect, respectively.^30^ The secondary outcomes underwent exploratory analysis to allow for a comprehensive examination of the treatment effects. For binary outcomes, differences and their 95% CIs were calculated using the Newcombe method.^31^ The numbers and frequencies of adverse events are described.

The robustness of the main results was confirmed through per-protocol set analysis and multiple imputation to treat missing data as sensitivity analyses. For binary outcomes, missing data at a specific time point were handled as a nonresponse at that time point (nonresponse imputation). Subgroup analyses were performed using MMRM based on age, OAB severity, pharmacotherapy status, incontinence (yes vs no), HADS anxiety score, and HADS depression score. Statistical analyses were performed with SAS, version 9.4 (SAS Institute Inc) and R, version 4.3.1 (R Foundation for Statistical Computing) software.

## Results

### Participants

**Figure 1.**
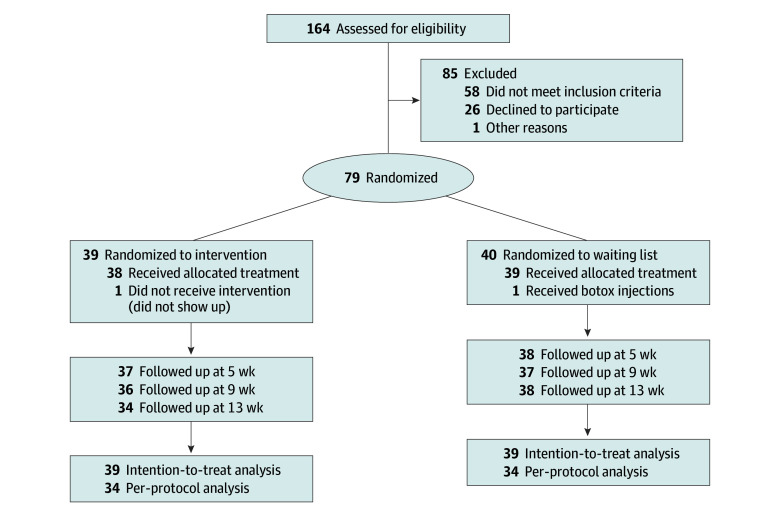
CONSORT Diagram of Trial Participation

A total of 164 women were invited to participate in the study, and 79 underwent randomization (Figure 1). Of the 79 randomized participants, 1 in the intervention group and 1 in the control group dropped out of the allocated intervention, and 5 in the intervention group and 2 in the control group dropped out of the primary outcome assessment at week 13. Only 1 in the intervention group participated in the online sessions. Table 1 summarizes the participant characteristics at baseline. In the intervention and control groups, respectively, the mean (SD) age was 63.5 (14.6) years and 63.5 (12.9) years, and OAB was moderate for 36 participants (92.3%) and 35 participants (87.5%).

**Table 1.** Participant Baseline Characteristics by Treatment Group

Baseline characteristic	No. (%)	
	Multicomponent intervention (n = 39)	Waiting list control (n = 40)
Age, mean (SD), y	63.5 (14.6)	63.5 (12.9)
BMI, mean (SD)	22.0 (3.1)	22.7 (5.0)
Marital status		
Single	4 (10.3)	6 (15.0)
Married	27 (69.2)	29 (72.5)
Divorced	2 (5.1)	3 (7.5)
Widowed	6 (15.4)	2 (5.0)
Parity and childbirth	31 (79.5)	32 (80.0)
Menopause	25 (64.1)	26 (65.0)
Education level		
Junior high school	0	3 (7.5)
High school	13 (33.3)	10 (25.0)
Vocational school	6 (15.4)	3 (7.5)
Junior college	12 (30.8)	10 (25.0)
University	8 (20.5)	12 (30.0)
Graduate school	0	2 (5.0)
Employment status		
Full-time	5 (12.8)	8 (20.0)
Part-time	10 (25.6)	11 (27.5)
Unemployed	0	2 (5.0)
Homemaker	16 (41.0)	15 (37.5)
Retirement	8 (20.5)	4 (10.0)
OAB severity		
Moderate	36 (92.3)	35 (87.5)
Severe	3 (7.7)	5 (12.5)
Duration of OAB, mean (SD), y	9.0 (9.3)	7.2 (9.2)
OAB medication		
No prior treatment	14 (35.9)	16 (40.0)
Past treatment	9 (23.1)	9 (22.5)
Current treatment	16 (41.0)	15 (37.5)
Mirabegron	11 (28.2)	11 (27.5)
Vibegron	2 (5.1)	3 (7.5)
Solifenacin	2 (5.1)	3 (7.5)
Imidafenacin	2 (5.1)	0
Fesoterodine	1 (2.6)	0
Other^a^	1 (2.6)	1 (2.5)
Comorbidities		
Hypertension	10 (25.6)	12 (30.0)
Hyperlipidemia	4 (10.3)	8 (20.0)
Heart disease	3 (7.7)	3 (7.5)
Diabetes mellitus	3 (7.7)	1 (2.5)
Gynecologic disease	3 (7.7)	1 (2.5)
Stroke	3 (7.7)	1 (2.5)
Cancer	2 (5.1)	3 (7.5)
Neurologic disease	0	0
Other^b^	9 (23.1)	9 (22.5)
Medication for other diseases		
Antihypertensive	11 (28.2)	16 (40.0)
Anticholesterol	6 (15.4)	12 (30.0)
Proton pump inhibitor	5 (12.8)	4 (10.0)
Antiplatelet drug	3 (7.7)	3 (7.5)
Insulin	2 (5.1)	1 (2.5)
Diuretic	2 (5.1)	1 (2.5)
Other^c^	6 (15.4)	9 (22.5)
Institution		
University hospital	4 (10.3)	3 (7.5)
General hospital A	5 (12.8)	6 (15.0)
General hospital B	3 (7.7)	4 (10.0)
Private clinic	27 (69.2)	27 (67.5)

Abbreviations: BMI, body mass index (weight in kilograms divided by height in meters squared); OAB, overactive bladder.

^a^ No further information was provided if other was selected.

^b^ Includes atrial fibrillation, hyperthymia, irritable bowel syndrome, osteoporosis, polymyalgia rheumatica, reflux esophagitis, and rheumatoid arthritis.

^c^ Analgesic, antiallergic, antiestrogen, antirheumatic, antithyroid, oral contraceptive, osteoporosis, ramosetron, and sleeping drugs.

### Primary Outcome

**Figure 2.**
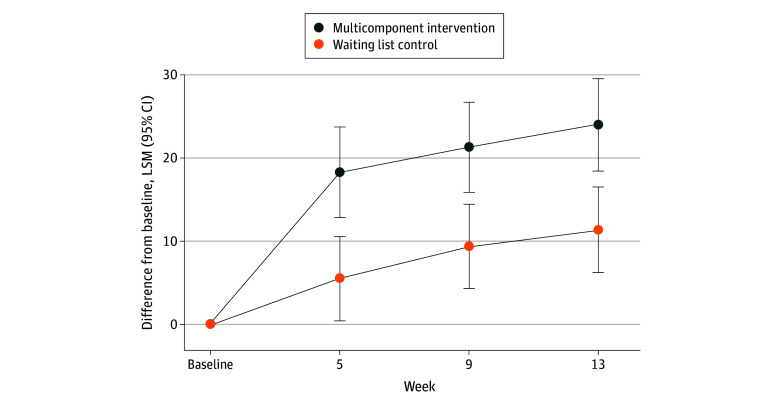
Least-Squares Mean (LSM) Changes in Overactive Bladder Questionnaire Scores From Baseline Over Time by Treatment Group

Figure 2 and Table 2 show the LSM changes from baseline at each assessment time for the HRQOL total scores of the OAB-q. The change in HRQOL total score from baseline to week 13 was 23.9 points (95% CI, 18.4-29.5 points) in the intervention group and 11.3 points (95% CI, 6.2-16.4 points) in the waiting list group. At week 5, immediately after the intervention, the HRQOL total scores of the OAB-q was 12.8 points (95% CI, 7.0-18.6 points; *P* < .001) higher in the intervention group than in the control group. The difference was maintained during the follow-up at week 9 (between-group difference estimate, 11.9 points; 95% CI, 6.1-17.7 points; *P* < .001) and week 13 (between-group difference estimate, 12.6 points; 95% CI, 6.6-18.6 points; *P* < .001) in the intervention group compared with the control group. Subgroup analysis findings suggested that the multicomponent intervention may be less effective in some subsets, such as older participants, those with no prior treatment, and those with anxiety (eTable 1 in Supplement 2). However, we found no definitive evidence of treatment effect modification, as this study was not designed to detect such heterogeneity. The group differences in the per-protocol population (eTable 2 in Supplement 2) and in the multiple imputation methods (eTable 3 in Supplement 2) were consistent with those of the intention-to-treat population. Among those who completed follow-up, 76.4% (26 of 34) of intervention group participants showed at least a minimally important improvement compared with 55.3% (21 of 38) of control group participants. Using the estimated LSM difference and pooled SD, the SMD was 0.82 (95% CI, 0.33-1.31).

**Table 2.** HRQOL Total Scores of the Overactive Bladder Questionnaire

Time point	Multicomponent intervention (n = 39)			Waiting list control (n = 40)			Comparison between groups		
	No. of participants	HRQOL score, points		No. of participants	HRQOL score, points		Difference in LSM changes (95% CI), points	*P* value	SMD (95% CI), points^a^
		Mean (SD)	LSM change (95% CI)		Mean (SD)	LSM change (95% CI)			
Baseline	39	60.6 (16.2)	NA	40	66.0 (16.9)	NA	NA		NA
5 wk	37	80.7 (12.6)	18.3 (12.9-23.7)	38	71.2 (19.2)	5.5 (0.4-10.6)	12.8 (7.0-18.6)	<.001	0.93 (0.44-1.41)
9 wk	36	84.2 (10.2)	21.3 (15.9-26.7)	37	74.7 (18.6)	9.4 (4.3-14.4)	11.9 (6.1-17.7)	<.001	0.87 (0.38-1.36)
13 wk	34	87.6 (8.6)	23.9 (18.4-29.5)	38	77.0 (18.4)	11.3 (6.2-16.4)	12.6 (6.6-18.6)	<.001	0.82 (0.33-1.31)

Abbreviations: HRQOL, health-related quality of life; LSM, least-squares mean; NA, not applicable; SMD, standardized mean difference.

^a^ The SMD was calculated by dividing the mean difference by the pooled SD. The mean difference was obtained from the difference in LSM changes, and the pooled SD was calculated from the SDs of the change scores between baseline and each time point in both the cognitive behavior therapy and waiting list groups.

### Secondary Outcomes

eTables 4 to 6 in Supplement 2 show a descriptive summary and comparisons between the 2 groups for the secondary outcomes. All secondary outcomes, except total OABSS, KHQ personal relations, KHQ severity measures, EuroQol 5-dimensional and 5-level questionnaire, and urge incontinence frequency, suggested superiority of the multicomponent intervention over the waiting list at 13 weeks (eTable 4 in Supplement 2). Patient-reported improvement and satisfaction were better in the intervention group than in the control group (eTable 5 in Supplement 2). Most participants in both groups had no change in the pharmacotherapy during the trial, but more participants stopped drug treatment in the intervention group (eTable 6 in Supplement 2).

### Adverse Events

One unexpected serious adverse event (hospitalization due to dehydration) in the intervention group was reported to the medical ethics committee and was determined to be not known to be related to the intervention. Another 7 adverse events were reported (4 in the intervention group and 3 in the control group). The breakdown of adverse events was as follows: acute cystitis (3 in the intervention group and 2 in the control group), herpes zoster infection (1 in the intervention group), and headache (1 in the control group).

## Discussion

The study findings demonstrate the efficacy of a multicomponent intervention with cognitive components for women with moderate to severe OAB. We observed improvements in disease-specific HRQOL over time (as measured by OAB-q scores), with a significant effect size and high feasibility and minimal side effects. Moreover, the effect size was large enough to achieve a clinically meaningful differentiation. The results were nearly consistent across sensitivity analyses, demonstrating the robustness of the results.

This RCT is the first to our knowledge to evaluate the efficacy of a multicomponent intervention with cognitive components for OAB in women, especially with the novelty of using the CBT model in educational sessions and exposure-based bladder training as a graded exposure task. The effect size was sufficient and comparable with that of conventional behavioral therapy reported in the previous network meta-analysis.^11^ Although 2 systematic reviews have reported on the cognitive components of behavioral therapy for UUI or OAB so far, they did not include studies that involved cognitive intervention.^17,18^ The first review considered behavioral therapy without a cognitive component.^17^ In contrast, the second review focused on behavioral therapy with cognitive components and included 5 studies; however, none of the included studies strictly adhered to CBT principles.^18^ Despite the lack of studies that explicitly included cognitive intervention as a treatment, both reviews emphasized the importance of incorporating cognitive components into behavioral therapy and suggest that cognitive approaches may be effective.

There are several hypotheses regarding the effect of the cognitive approach for OAB. A previous case series study reported that urinary urgency may be triggered not only by sensation but also by cognitive processes such as “knowledge of the time of the last void, anticipated time to the next void, proximity of toilets, risk assessment or habituated behavior.”^15^^(p1756)^ Other previous functional brain imaging studies have suggested that sensory urgency has an affective component and that the brain areas involved are similar to those activated by other unpleasant sensations, such as pain, dyspnea, pruritus, and the urge to cough.^32,33^ These studies suggested that cognitive factors may contribute substantially to the severity of OAB symptoms. Consequently, a cognitive approach may prove essential in modifying both symptom perception and behavioral responses. Although we found that a multicomponent intervention with cognitive components improves micturition and urgency symptoms, these were not substantial enough to show a significant change in total OABSS. It is important to note that improvements in HRQOL do not always correspond to changes in the total OABSS.

### Strengths and Limitations

This study has several strengths. First, it was conducted according to a steady, step-by-step plan. We conducted a pilot study to establish a multicomponent intervention with cognitive components for women with OAB, and based on the results of that study, we developed a treatment manual and published a protocol before initiating this main trial. Second, this trial was designed using a robust process that achieved high internal validity by minimizing the biases associated with an RCT, including appropriate random assignment and low rates of dropout from the intervention and outcome measures. In addition, the robustness of our results was further supported by prespecified subgroup and sensitivity analyses. The use of MMRM in the longitudinal data analysis allowed for a more accurate estimation of effects, even in the presence of missing data based on the assumption of missing at random. Third, the multicomponent intervention was conducted according to the established manual, which ensured the quality of the interventions and future transferability. Such manual development ensures the reproducibility of the multicomponent intervention, and the external validity can be verified by using it in future clinical trials.

This study also has several limitations. First, the trial was open label, and both participants and therapists were aware of the intervention group. This design may introduce a detection bias in favor of the intervention in the assessment of patient-reported outcomes, and caution should be exercised to avoid overestimating the results due to bias.^34^ However, masking is generally challenging in behavioral therapy intervention trials. Superiority of the multicomponent intervention was noted across most of the secondary outcomes, including the hard outcomes of voiding frequency and urgency frequency. Second, although we used a waiting list control group to compare the active intervention’s efficacy and provided participants on the waiting list a chance for intervention later for ethical reasons, such trial designs are known to show greater differences between the intervention and control groups.^35^ The possibility of overestimating the effect should be noted in this study design. Third, the participants included those taking and not taking OAB medication at baseline. Although the subgroup analysis did not show any evidence of treatment effect modification, it is possible that the effect of the multicomponent intervention is not uniform for such a heterogeneous target population. Fourth, the sample size was smaller than what we had initially planned due to the COVID-19 pandemic and the shortage of funding. However, the effect size turned out to be much larger than originally anticipated, and it should be acknowledged that the sample size calculation in the protocol was premature. Fifth, this multicomponent intervention was conducted by a single expert clinician, which could lead to a lack of generalizability of the study results.

## Conclusions

In this RCT, a multicomponent intervention improved HRQOL for women with moderate to severe OAB and demonstrated high feasibility. These findings suggest that a multicomponent intervention with cognitive components may be an effective treatment option for women with OAB.

## Notes

Supplement 1.
Trial Protocol

Supplement 2. **eTable 1.** HRQL Total Scores of OAB-q at Baseline, 5, 9, and 13 Weeks (Subgroup Analysis) **eTable 2.** HRQL Total Scores of OAB-q at Baseline, 5, 9, and 13 Weeks (Per-Protocol Set Analysis) **eTable 3.** HRQL Total Scores of OAB-q at Baseline, 5, 9, and 13 Weeks (Multiple Imputation) **eTable 4.** Secondary Outcomes at Baseline, 5, 9, and 13 Weeks **eTable 5.** Secondary Outcomes at Baseline, 5, 9, and 13 Weeks (PGI) **eTable 6.** Secondary Outcomes at Baseline, 5, 9, and 13 Weeks (Change in Pharmacotherapy) **eFigure 1.** Cognitive Behavior Therapy Model for Overactive Bladder **eFigure 2.** Example of Exposure Technique Using a Graded Exposure Task

Supplement 3.
Data Sharing Statement
