# Supplementary material for: Multicomponent Intervention for Overactive Bladder in Women: A Randomized Clinical Trial
Source: JAMA Netw Open. 2024 Mar 13;7(3):e241784. doi: 10.1001/jamanetworkopen.2024.1784 (PMC10938174; doi:10.1001/jamanetworkopen.2024.1784)
Supplement: Supplement 1. — Trial Protocol [file jamanetwopen-e241784-s001.pdf]

Efficacy of Cognitive Behavioral Therapy for Overactive Bladder in Women: A Randomized Controlled Trial

Trial Protocol

Principal Investigator: Takashi Kobayashi[1]

Sub-Investigators: Satoshi Funada[1], Shusuke Akamatsu[1], Takayuki Goto[1], Kentaro Ueno[2], Ryuji Uozumi[2], Kentaro Ichioka[3], Yumiko Nakagawa[4], Takehiko Segawa[5], Tatsuo Akechi[6], ToshiA. Furukawa[7]

Affiliations:

1. Department of Urology, Kyoto University Graduate School of Medicine, Kyoto, Japan
2. Department of Biomedical Statistics and Bioinformatics, Kyoto University Graduate School of Medicine, Kyoto, Japan
3. Ichioka Urological Clinic, Kyoto, Japan
4. Department of Urology, Kyoto City Hospital, Kyoto, Japan
5. Kyoto Min-Iren Asukai Hospital
6. Department of Psychiatry, Nagoya City University Graduate School of Medical Sciences, Aichi, Japan.
7. Department of Health Promotion and Human Behavior, Kyoto University School of Public Health, Kyoto, Japan

Study sites: Kyoto University Hospital, Kyoto Municipal Hospital, Kyoto Civil Medical Federation Asukai Hospital, Ichioka Urology Clinic

Ver. 1.1 October 9, 2019
Ver. 1.2 October 17, 2019
Ver. 1.3 November 4, 2019
Ver. 1.4 November 12, 2019
Ver. 1.5 November 15, 2019
Ver. 1.6 November 25, 2019
Ver. 1.7 December 2, 2019
Ver. 1.8 December 21, 2019
Ver. 1.9 January 16, 2020
Ver. 2.1 June 10, 2020

Ver. 2.2 June 12, 2020
Ver. 2.3 June 30, 2020
Ver. 2.4 November 15, 2021
Ver. 2.5 November 30, 2021
Ver. 2.6 March 1, 2022
Ver. 2.6 March 3, 2022

Outline of Clinical Trial

0.1 Objective.

To evaluate the efficacy of a cognitive behavioral therapy (CBT) program for female patients with overactive bladder (OAB).

0.2 Study Design

Open-label, parallel-group, multicenter randomized controlled trial

0.3 Flowchart of the study

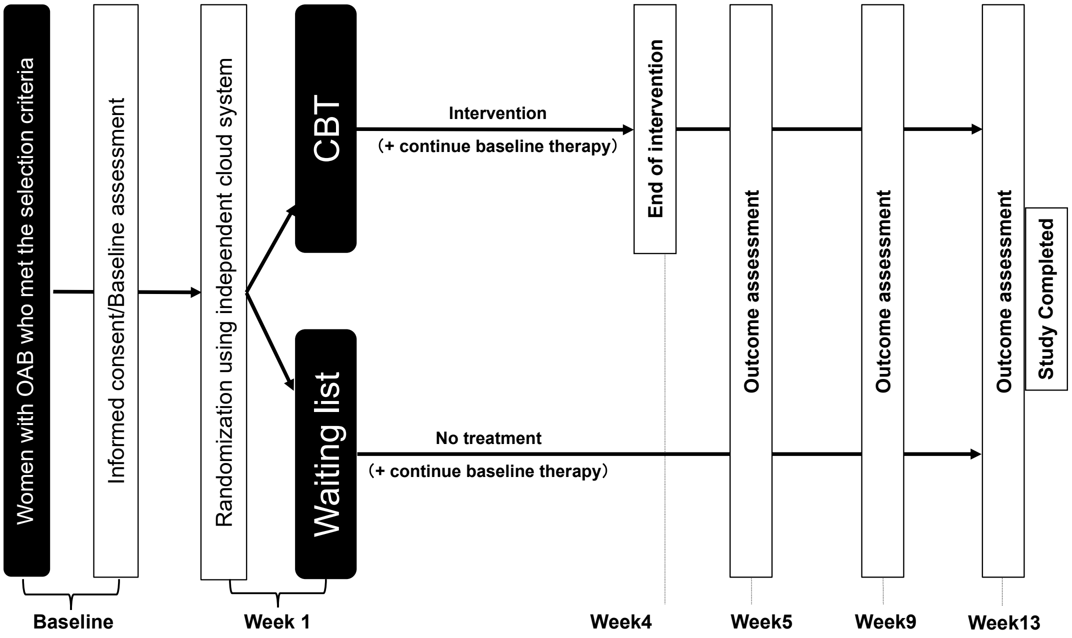

0.4 Study participants

Women aged 20 to 80 years who were diagnosed with moderate or severe OAB at the time of their first visit or during their first visit to the urology outpatient department of a general hospital or urology clinic (1). The patients will be included regardless of whether or not they are receiving medical treatment for OAB.

0.5 Method of intervention

Intervention group: CBT, 30 minutes per session, once a week for a total of 4 treatment sessions. If the patient is on medical treatment, it will be given in conjunction with medical treatment. Online intervention using a web conferencing application will be

provided for those who wish to use.

Control group: No additional treatment during the study period. If on medical treatment, participants will continue the baseline medical treatment only. CBT will be provided at the end of the study. If desired, CBT will be conducted online using a web conferencing application.

0.6 Outcomes

Primary outcome:

- Health-related quality of life (HRQoL) total score of Overactive Bladder Questionnaire (OAB-q) (2)

Secondary outcomes:

- The other subscales of OAB-q (2)
- King Health Questionnaire (KHQ) (3)
- Overactive bladder symptom score (OABSS) (1)
- Patient Global Impression-Improvement (PGI-I) (4)
- Patient Global Impression-Severity (PGI-S) (4)
- EQ-5D-5L (5)
- Hospital Anxiety and Depression Scale (HADS) (6)
- Frequency volume chart (FVC)
- Patient Satisfaction
- Treatment compliance
- Dropout rate
- Change in medication (change, addition, or discontinuation)
- Incidence of adverse events

0.7 Target number of patients

150 patients

0.8 Study duration

Three years from the date of approval

1. Background

1.1 Definition of OAB

OAB was defined by the International Continence Society (ICS) in 2002 as a symptom syndrome in which urinary urgency is essential, usually accompanied by urinary frequency and nocturia, and urge incontinence is not essential (7). It is generally recognized as a disease concept consisting of symptoms such as urinary urgency, frequent urination, nocturia, and urge urinary incontinence. According to the guidelines for OAB, urinary urgency is a sudden, unbearable urge to urinate that is difficult to explain and differs from the normal urge to urinate (8). Urge urinary incontinence is defined as an involuntary leakage of urine at the same time as or immediately after the urge to urinate (8).

1.2. Prevalence of OAB

The epidemiology of OAB in Japan was reported in a large epidemiological survey in 2002 (9). In this epidemiological survey, 10,096 men and women aged 40 years or older were randomly sampled from 75 sites nationwide, and the prevalence of OAB in women was 10.8%, with a tendency to increase with age. The number is expected to increase further in the aging society.

1.3. Impact of OAB on quality of life

OAB is a chronic condition that does not cause death by itself. However, complete cure is difficult, and the more severe the symptoms, the more impaired the quality of life (QoL) is known to be (10).

1.4. Impact of OAB on Medical Economics

The total cost related to OAB in Japan, calculated based on the prevalence rate in a large-scale epidemiological survey in 2002, was 956.2 billion yen per year (11). The breakdown was 180.9 billion yen (19%) for the treatment of OAB (including 159.1 billion yen for drug treatment), 92.7 billion yen (6%) for the treatment of OAB comorbidities, 28.7 billion yen (3%) for urinary incontinence-related costs, and 684.6 billion yen (72%) for labor losses. Furthermore, with or without treatment, the cost was estimated at 112,000 yen per symptomatic patient. With the super-aging of the population, its impact on the healthcare economy is expected to increase.

1.5. Current status of OAB treatment

The 2015 revised guidelines for the treatment of OAB describe behavioral,

pharmacologic, neuromodulation, and surgical therapies for the treatment of OAB. The most commonly used treatment for OAB is pharmacotherapy, especially anticholinergic drugs, the efficacy of which is well-established (8). However, there are many reports of side effects associated with systemic muscarinic receptor blockade, including dry mouth and constipation, and a systematic review reported that 43-83% of patients discontinue medication within one month (12). Recently, a large case-control study reported an association between anticholinergics and the development of dementia (odds ratio 1.11, 95% confidence interval 1.08-1.14) (13). Neuromodulation therapy and surgical treatment methods are invasive treatments, and the number of facilities that perform them is limited.

1.6 Behavioral Therapy

Behavioral therapy improves bladder control by helping patients change their behavior through learning urinary habits and prevention methods for urinary urgency. Specifically, it consists of lifestyle modifications, bladder training, and pelvic floor muscle training. Lifestyle modifications aim to improve aggravating factors of OAB, such as weight loss, limitation of drinking, limitation of alcohol consumption, and smoking cessation. Bladder training aims to improve symptoms by prolonging the voiding interval through making the patient hold their urine. Pelvic floor muscle training is a physical therapy program that strengthens the contractility of the pelvic floor muscles to improve symptoms.

Because of its safety, the guidelines of the American Urological Association list behavioral therapy as the first-line treatment (14). However, treatment programs are not well-established. Especially in Japan, it is not widely used because it has not been determined who, where, and how it should be conducted, and it is not reimbursed by medical costs.

1.7 Cognitive behavioral therapy (CBT)

CBT is a form of psychotherapy developed by Aaron T. Beck in 1963 from cognitive therapy for depression (16). Since then, it has been widely used for anxiety disorders such as panic disorder and obsessive-compulsive disorder, as well as other psychiatric disorders. The theory of CBT is based on the fact that cognition (the way one thinks and perceives things) affects emotions and behavior, which in turn manifest themselves as physical symptoms. External stress distorts cognition, which in turn affects emotions and behavior, causing physical symptoms, which in turn distort cognition further, creating a vicious cycle. This leads to disease. CBT corrects distorted cognition and

behavior and cures symptoms.

CBT is characterized by:

- Aiming to solve the problem in the here and now.
- The therapist and the research participant working together to set goals and determine a treatment plan (cooperative positivism).
- Both sessions and programs being structured.
- Treatment following a cognitive behavioral model.

In addition to psychiatric disorders, it has been reported to be effective for other non-psychiatric disorders such as chronic pain (17) and irritable bowel syndrome (18). OAB can also be addressed using the cognitive behavioral model (see figure below), and we believe that a CBT approach that includes the aforementioned behavioral therapy may also be effective.

Cognitive behavioral model for OAB and target of each technique.

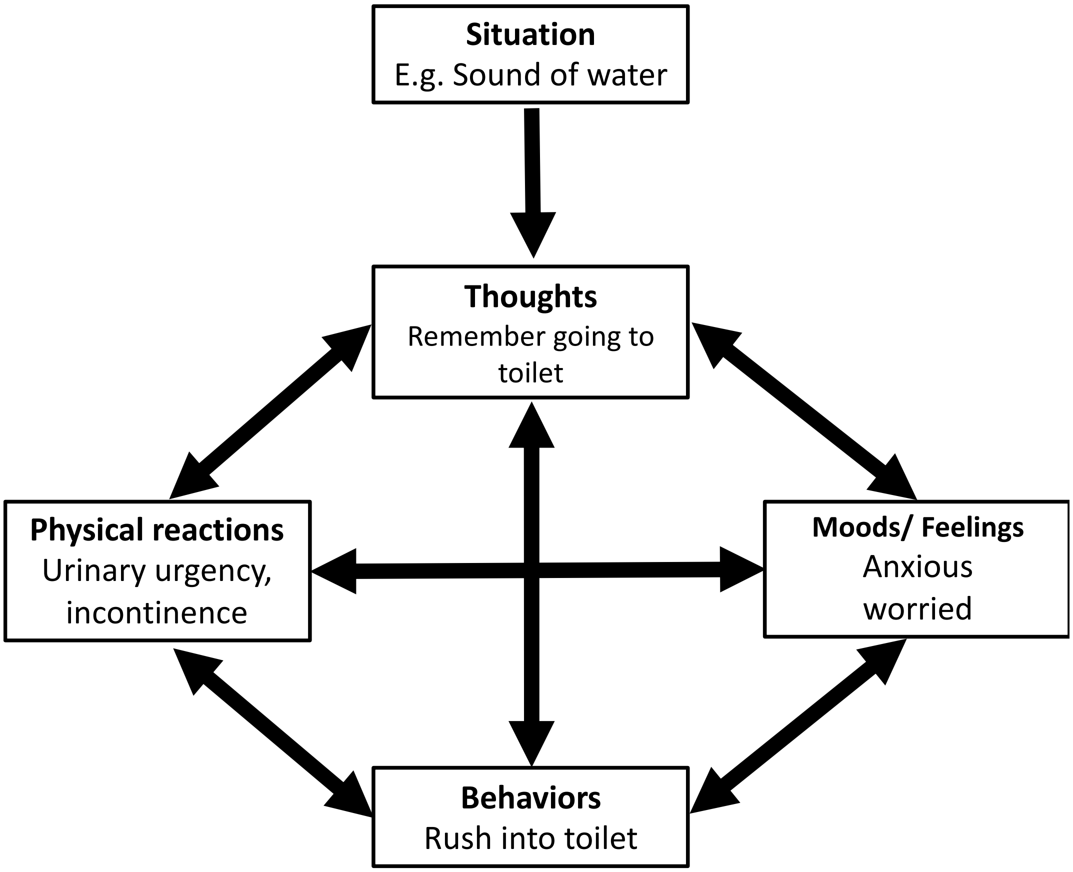

1.8. About the pilot study

To evaluate the feasibility of CBT for drug-resistant OAB, a pilot study of 10 cases was conducted from April 2019 to October 2019 (approved by the Kyoto University Medical Ethics Committee, receipt number: C1423, approval date: January 16, 2019, "Conventional A Single-Group, Uncontrolled Intervention Study of the Feasibility and Efficacy of a Combined Behavioral Therapy and Psychological Intervention Program for Drug-Resistant Overactive Bladder"). There was one participant who dropped out of the study due to an adverse event (AE), but it was not related to the study. Other participants completed the study without AEs. Although statistical tests were not performed, both primary and secondary outcomes improved before and after the intervention. Based on the results of the pilot study, we concluded that it was worthwhile to evaluate the efficacy of CBT for OAB and decided to design this study.

2. Purpose

The purpose of this study is to establish CBT for OAB based on conventional behavioral therapies and to evaluate its efficacy in women with moderate to severe OAB in a randomized controlled trial.

3. Study design

Open-label, parallel-group, multicenter, 1:1 randomized controlled trial

4. Selection of participants

4.1. Institutions

Department of Urology, Kyoto University Hospital

Department of Urology, Kyoto City Hospital

Department of Urology, Asukai Hospital, Kyoto Civil Medical Federation Ichioka Urology Clinic

4.2 Inclusion and exclusion criteria

Inclusion criteria:

(1) Women between 20-80-year-old;

(2) Participants not having undergone any OAB treatment for more than 6 weeks or participants taking pharmacotherapy lasting more than 6 weeks for OAB;

(3) Participants diagnosed with OAB based on their OAB symptom score (OABSS)* (a total OABSS score ≥3, with an urgency score ≥2), with the total score being higher than 6 (OABSS; 6 to 11 points indicate moderate severity, 12 to 15 points indicate moderate severity) (1);

(4) An Eastern Cooperative Oncology Group (ECOG) Performance Status of Grade 0; and

(5) Participants who are able to understand explanations and sign a written informed consent.

*Overactive Bladder Symptom Score (OABSS):

It is a symptom score for OAB developed in Japan and used worldwide for diagnosis and severity assessment (1). A urinary urgency score (Question 3) of 2 or higher and a total score of 3 or higher are considered a diagnosis of OAB. Regarding severity, a total score of 5 or less is considered mild, a score of 6-11 moderate, and a score of 12 or more severe.

Exclusion criteria

(1) Abnormalities around the bladder (e.g., bladder cancer, bladder calculus, interstitial cystitis, endometriosis);
(2) Urinary tract infection;
(3) Surgery for urinary incontinence;
(4) Pregnancy;
(5) Disability to understand Japanese;
(6) Depression;
(7) Dementia; and
(8) Participants judged otherwise unsuitable for participation by the researchers.

4.3 Selection of Study Participants

During recruitment (see 23), individuals interested in participating in this study will be asked to visit a research collaborating facility and undergo a general medical examination for OAB. The general examination for OAB aims to determine the diagnosis and severity of the condition through a questionnaire-based interview and ultrasound to rule out abnormalities around the bladder. Those who meet the inclusion and exclusion criteria will be provided with an explanation of the study, and those who give their consent will be included in the study.

Intervention

5.1 Intervention CBT program used in this study

In addition to the conventional behavioral therapy that has been used for OAB (3, 4), CBT techniques (1, 2, 5, 6) known to be particularly effective for OAB will be employed.

(1) Participant education

A general explanation of OAB and CBT will be provided, and short- and long-term goals for undergoing this treatment will be set. This will also foster a cooperative relationship between the participant and the treatment interventionist.

(2) Self-monitoring

The participant will maintain a urinary diary (FVC) to monitor their current status independently. By continuing this practice not only during the treatment evaluation but also throughout the intervention period, the patient can observe the changes in their urinary status. This will help them identify and overcome their own challenges, and the visible improvement will enhance their self-efficacy.

(3) Lifestyle modifications

Identify lifestyle habits that may be risk factors for OAB (excessive water intake, caffeine consumption, alcohol consumption) and establish strategies to improve them.

(4) Pelvic floor muscle training

Weak pelvic floor muscles are considered one of the causes of OAB. In conjunction with proper posture and breathing techniques, increase pelvic floor muscle strength through repeated awareness and contraction/relaxation exercises.

(5) Exposure

While the urge to urinate can be avoided by emptying the bladder frequently, the act of frequent urination actually promotes the urge to urinate, leading to a vicious cycle. Exposure (endurance) to the uncomfortable situation (urge to urinate) is encouraged to increase tolerance to the urge (to extend the interval between urinations). A step-by-step schedule should be planned, and the urination interval should be gradually increased by consistently following the schedule.

(6) Relapse prevention

Review the series of sessions and compare the condition before and after treatment. List the positive and negative aspects and help the participants solve problems independently, such as how to continue the treatment in the future or what to do in case of symptom exacerbation.

Using these six techniques, interventions that may be effective for OAB are selected, and the therapist and the research participant collaborate to set treatment goals and assign homework for each session. Each session consists of 30 minutes and includes (1) introduction, (2) agenda, and (3) summary. The overall treatment will comprise of (1) an introduction phase (1 session), (2) a practice phase (2 sessions), and (3) a relapse prevention phase (1 session), totaling 4 sessions. As a basic rule, interventions will be conducted every week, but a maximum interval of three weeks will be allowed (an interval of more than three weeks will be considered a deviation from the intervention). If the patient is already on oral therapy, the CBT should be coordinated with the oral therapy.

Due to concerns about the risk of novel coronavirus transmission, the intervention will be conducted online using a web conferencing application for those who wish to participate. The web conferencing application chosen for this purpose is Zoom, a free web conferencing service provided by Zoom Video Communications, Inc. of the United States (https://zoom.us/). A reminder email containing the Zoom appointment details will be sent to the participants one day before the intervention. The intervention will be conducted using Zoom, following the same treatment intervention program as the in-person sessions.

Brief summary of CBT manual for drug-resistant OAB.

Sessions	Description
1	**Education**<br>Education about voiding function, OAB and CBT<br>**Life-style modification**<br>Check drinking water and coffee, taking exercise<br>Modify bad life-styles<br>**PFMT**<br>Teach by using photos and a yoga-ball<br>Ten minutes/session and more then 3 sessions each day<br>**Review and homework**
2	**Review session 1 and homework**<br>**PFMT**<br>**Exposure**<br>Set a goal and several steps to achieve the goal<br>**Review and homework**
3	**Review session 2 and homework**<br>**PFMT**<br>**Exposure**<br>Set the next steps and goal<br>**Review and Give homework**
4	**Review session 3 and homework**<br>**Relapse prevention**<br>Plan the future's goal and encourage to continue the techniques<br>**Total review**

5.2. Therapist

The treatment therapist in this study shall be qualified as a specialists and supervisors certified by the Japanese Urological Association, with a minimum of 10 years of clinical experience. Additionally, participation in the CBT workshop, a training program conducted by the Ministry of Health, Labor, and Welfare, is mandatory.

5.3 Assurance of Quality of Intervention

To ensure the quality of interventions, a review committee comprising urologists, obstetricians/gynecologists, psychiatrists, clinical psychologists, and physical therapists was established, and a treatment manual was developed. The therapist will conduct interventions in accordance with this manual. A checklist is prepared for each session to verify the achievement of each item.

5.4. Treatment of the control group

CBT will not be administered during the study period. If the patient is receiving a drug treatment, only the baseline treatment will be continued. CBT will be provided after the completion of the study. Online CBT using a web conferencing application will be conducted for those who wish to participate.

6. Data collection

6.1 Baseline characteristics

Age, height, weight, medical history, childbearing experience, menopause, highest education level completed, marital status, occupational history, and history of dysuria treatment will be assessed using a questionnaire at baseline.

6.2. Outcomes

Primary outcome:

- Overactive Bladder Questionnaire (OAB-q) HRQoL total score (2)

This is a QOL self-reported questionnaire specific to OAB. It consists of questions regarding the degree of distress and QOL due to symptoms. Week 13 (end of follow-up) is the primary assessment point. The HRQoL total score of the OAB-q is calculated on a 100-point scale as follows: Minimum Clinical Important Difference (MCID) is 10 points (20).

[150 - (Question Score 9+10+11+12+13+14+15+16+17+18+19+20+21+22+23+24+25+26+27+28+29+30+31+32+33)]/125 * 100

Secondary outcomes:

- OAB-q subscales other than HRQoL total scores (2)

The subscales other than the HRQoL total score are calculated as follows.

- Symptom bother: [(Question score 1+2+3+4+4+5+6+7+8)-8]/40 * 100
- Coping behaviors: [48 -(question score 9+11+16+21+22+26+32+33)]/40 * 100
- Concern/worry: [42 -(question score 12+13+14+19+23+25+29)]/35 * 100
- Sleep: [30 -(question score 10+15+17+24+30)]/25 * 100
- Social interaction: [30 -(question score 18+20+27+28+31)]/25 * 100
- Overactive bladder symptom score (OABSS)(1)

This is a self-administered questionnaire symptom score for OAB developed in Japan and used for diagnosis and severity assessment. Regarding severity, a total score of 5 or less is judged as mild, 6-11 as moderate, and 12 or more as severe.

- King Health Questionnaire (KHQ) (3)

A QoL self-administered questionnaire specific to urinary incontinence, consisting of 19 questions in 8 domains, which has also been validated for OAB.

- General health perception: [(Q1 score - 1]/4 * 100
- Incontinence impact: [(Q2 score -1]/3 * 100
- Role limitations: [(Q3a+Q3b score -2]/6 * 100
- Physical limitations: [(Q4a+Q4b score -2]/6 * 100
- Social limitations: [(Q4c+Q4d+Q5c score -3]/9 * 100 when 1≤ Q5c

When Q5c = 0, [(Q4c+Q4d+Q5c score -2]/6 * 100

- Personal relationships: when 2≤ Q5a+Q5b, [(Q5a+Q5b score -2]/6 * 100

When Q5a+Q5b = 1, [(Q5a+Q5b score - 1]/3 * 100

When Q5a+Q5b = 0, treat as missing values

- Emotions: [(score of Q6a+Q6b+Q6c - 3]/9 * 100
- Sleep/energy: [(Q7a+ Q7b score -2]/6 * 100
- Severity measures: [(Q8a+ Q8b+ Q8c+ Q8d+ Q8e score -5]/15 * 100
- Patient Global Impression-Improvement (PGI-I) (4)

A multi-item option to evaluate the general improvement score of symptoms using a 7-point Likert scale self-administered questionnaire, with 1 to 2 defined as improvement, and a binary variable of with improvement and without improvement.

- Patient Global Impression-Severity (PGI-S) (4)

It is a multiple-choice instrument that evaluates the overall improvement score of symptoms using a 4-point Likert scale self-administered questionnaire. 1 to 2 is defined as improvement, and is evaluated with and without improvement as a binary variable.

- EQ-5D-5L (5)

A 5-question scale measuring HRQoL with a self-administered questionnaire.

- Hospital Anxiety and Depression Scale (HADS) (6)

A 14-item self-administered questionnaire developed to assess anxiety and depression in participants with physical symptoms. Anxiety and depression are calculated by summing the scores from a point distribution table. Scores of 0-7 indicate no anxiety or depression, scores of 8-10 are suspicious diagnoses, and scores of 11 or higher are confirmed diagnoses.

- Frequency voiding chart (FVC)

The following information is entered in a self-administered form: waking time, sleeping time, time of urination, amount of urination per time, frequency of urination, and presence or absence of urinary incontinence with a sense of urgency. Of these, the average number of urination, urinary incontinence, and urinary urgency frequency per 24 hours will be used as the evaluation items.

- Patient Satisfaction

A general improvement score of symptoms was used as a multiple choice to be evaluated using a self-administered questionnaire with a 5-point Likert scale. 1 to 2 is defined as satisfaction with treatment and is evaluated with a binary variable of satisfied or not satisfied.

- Change in medication (change, addition, or discontinuation)
- Treatment compliance

The degree of compliance with the techniques used in each session is evaluated.

Session 1	Self-monitoring
	Improvement of lifestyle
	Pelvic floor muscle exercises
Session 2	Self-Monitoring
	Lifestyle modification
	Pelvic floor muscle exercises
	Exposure
Session 3	Self-Monitoring
	Improvement of lifestyle
	Pelvic floor muscle exercises
	Exposure
Session 4	Self-Monitoring
	Improvement of lifestyle
	Pelvic floor muscle exercises
	Exposure

At the time of evaluation after the end of the intervention, the degree to which each technique is performed (compliance) will be assessed.

- Dropout rate

The percentage of assigned participants who drop out during the study will be calculated. There will be two types of dropouts in this study: intervention discontinuation and evaluation discontinuation (see "9. Discontinuation Criteria" for details).

6.3. Measurement methods

OAB-q, OABSS, KHQ, GIS, EQ-5D-5L, HADS, FVC, and treatment compliance before the start of CBT, at Week 5 (after treatment ends), Week 9 (4 weeks after treatment ends), and Week 13 (at the end of follow-up) of the paper or online-based registration system (REDCap*) Patients will be asked to complete a self-administered

questionnaire. Dropout rates and incidence of AEs will be confirmed by the therapist at each visit. Treatment feedback will be completed at the end of follow-up, either on paper or in a self-administered questionnaire in REDCap*. The same will be done for the study at all sites, and data will be collected online with security assured.

* REDCap is a data collection and management system developed by Vanderbilt University in the U.S. under the NIH Clinical Bridging Research Support Grant and distributed free of charge mainly to academia, and is used at 3090 facilities in 128 countries around the world. In Japan, Osaka City University provides the system; on April 1, 2017, the Department of Clinical Statistics, Graduate School of Medicine, Kyoto University contracted with Osaka City University to begin research use of the system. Under this contract, the data server is dedicated to Kyoto University, which is contracted by Osaka City University, and its management is entrusted to Osaka City University. The data server is equipped with complex security measures. Access to the server and system is restricted by user accounts and passwords issued to individuals, and all audit trails are recorded.

The schedule of this study.

	STUDY PERIOD							
	Enrolment	Allocation	Post-allocation			Post intervention	Follow-up	Close-out
TIMEPOINT:	0	1	2	3	4	5	9	13
ENROLMENT								
Eligible screen	X							
Informed consent	X							
Baseline characteristics	X							
Allocation		X						
INTERVENTION:								
Intervention group	X					X	X	X
Control group	X	X				X	X	X
ASSESMENTS:								
Primary outcome	X					X	X	X
Secondary outcome	X					X	X	X
Adverse Events		X	X	X	X	X	X	X
Drop out		X	X	X	X	X	X	X
Adherence		X	X	X	X			

7. Registration and random allocation

7.1. Enrollment procedure

Women aged 20-80 years who have been diagnosed with moderate to severe OAB at the time of their first visit or while attending a participating facility for the study will be screened through a general medical examination using a questionnaire and ultrasound. After confirming that the participants meet the eligibility criteria and do not meet the exclusion criteria, an explanation of the study will be provided using the study's explanatory consent document. The potential participants will be informed that participation is voluntary, they can withdraw even after participation, and that non-participation will not be detrimental to their treatment. Once consent is obtained for the explanation, the document will be signed. After receiving consent, basic information and OABSS scores will be entered into REDCap (see 6.3), and the participant will be registered. Each enrolled participant will be assigned an enrollment number by the site, and the enrollment number assigned by REDCap will be used to manage the data of enrolled participants for the entire study.

7.2. Random allocation procedure

Random allocation will be made 1:1 to the intervention or control group by the minimization method based on the basic information registered in REDCap and the assignment factors from OABSS on the computer. Allocation will be done by registration in the UMIN INDICE cloud system* (registration number: C00073) and using the algorithm in the system. During the first week of the intervention, the results

of the allocation will be communicated simultaneously to the therapists and the participants via an Internet screen. The intervention group will be informed that the intervention will start on the day of allocation, while the control group will be informed that no intervention will be given during the treatment period, and that the intervention will be given after the completion of the study.

*UMIN INDICE Cloud System (https://www.umin.ac.jp/indice/cloud.html)
This is a cloud system provided by UMIN that allows random assignment by the minimization method. If the difference between the two groups exceeds a certain level, the patients are assigned to the group with the lowest allocation. If the difference between the two groups is less than a certain level, random assignment is possible.

7.3. Allocation factors
(1) Age: 2 levels: 20-64 years old and 65-80 years old
(2) OAB severity: 2 levels: OABSS 6-11 points, OABSS 12-15 points
(3) Oral treatment: 3 levels: no prior treatment, prior treatment but more than 6 weeks have passed since the last dose, and currently on oral treatment.
(4) Institution: 4 levels (4 facilities)
(5) Intervention method: 2 levels: face-to-face, web conference
Age is considered the most significant risk factor for OAB, and the understanding of CBT may differ according to age, and the effectiveness of the intervention may also differ. We believe that OAB severity strongly influences intervention effectiveness. Intervention effectiveness may differ depending on whether CBT alone or in combination with oral medication is used. The backgrounds of the participants are expected to differ between facilities. For these reasons, age, OAB severity, presence of medical treatment, and facility were used as allocation factors.

7.4. Random allocation by minimization method
Each allocation factor will be summed, and if the difference between the two groups is greater than 2, the patient will be assigned to the allocation group with fewer allocation factors. If the difference between the two groups is less than 2, random assignment will be done to the randomly assigned group. The allocation factors will be entered into the UMIN INDICE cloud system in front of the therapist/participant or via online screen sharing during Week 1, and the allocation will be done on the spot.

8. Masking

Due to the nature of the intervention, this study cannot be masked to the therapist/participant. Statistical analysts will not be able to review the results until the end of the study. In addition, REDCap keeps a complete history, so the history can be traced for any evidence of confirmation of the results.
Masking: Statistical Analysts
Not Masking: Participants, Therapists, Outcome adjudicators

9. Discontinuation criteria

There will be two types of discontinuations from the study: intervention discontinuation and evaluation discontinuation.

9.1. Deviation from protocol treatment

Any of the following will be considered a deviation from the protocol treatment. In such a case, the participant has not dropped out of the study, and periodic evaluation will be conducted unless the participant withdraws consent for evaluation.
(1) If the participant has never received any intervention treatment
(2) When the intervention interval is more than 3 weeks.
(3) When the participant has received additional treatment for OAB at another hospital.
(4) When there are no valid data after the start of treatment.

9.2. Discontinuation of intervention

If any of the following conditions apply, the protocol treatment will be discontinued at the discretion of the physician in charge of the study. In such cases, the participant is not dropped from the study, and subsequent periodic evaluations will be conducted unless the participant withdraws consent for the evaluation.
(1) When the participant wishes to discontinue the intervention
(2) When a serious AE occurs and it is deemed difficult to continue the intervention
(3) When the physician in charge of the study determines that the harm of continuing the intervention outweighs the benefit for some reason, although no serious AE has occurred
(4) Other cases in which the investigator deems it inappropriate to continue the intervention.

9.3. Discontinuation of valuation

If a study participant withdraws consent for a periodic evaluation, no further follow-up will be conducted.

10. Reporting of adverse events and ensuring the safety of study participants

10.1 Definition of adverse events

Adverse events (AEs) are any unwanted or unintended signs or symptoms that occur in study participants, regardless of whether or not they are causally related to the intervention. For each AE, the severity, severity, transposition, and causal relationship to the intervention will be ascertained.

10.2 Measures to be taken and reporting procedures in case of AEs according to the Pharmaceutical Affairs Law

Since all oral treatments in this study are within the approved dosage and administration, the physician in charge will report to the MHLW if necessary, in accordance with "Article 77-4-2, Paragraph 2 of the Pharmaceutical Affairs Law" and "Kyoto University Graduate School of Medicine and Faculty of Medicine and Kyoto University Hospital Medical Ethics Committee Operating Procedures11".

10.3. Measures to be taken and procedures for reporting AEs according to the Ethical Guidelines for Clinical Research

In the event of a serious AE, the physician in charge will take necessary and appropriate measures to ensure the safety of the participant. According to the Ethical Guidelines for Clinical Research Q&A, what is a serious AE?

A) Deadly
B) Life-threatening
C) Requiring hospitalization or prolonged hospitalization for treatment
D) Results in permanent or significant disability or dysfunction
E) Inherited birth defects.

The term "serious AE" refers to the following. If a serious AE is suspected, within 48 hours, the physician in charge will evaluate whether or not the event constitutes a serious AE, regardless of whether or not it is causally related to the drug, in accordance with the AE Reporting Protocol. If it is determined to be a serious AE, the PI will report it to the Ethics Committee of the respective institution within 72 hours. The PI will also notify all investigators, who will take necessary action based on the information provided by the PI.

10.4 Anticipated adverse events

AEs associated with CBT will not occur in the pilot study and are not anticipated. When

taken concomitantly, anticholinergics or β3-adrenoceptor agonists are used. AEs associated with oral treatment include the following:

Anticholinergics

Main side effects: dry mouth and dry mouth, constipation, photophobia, blurred vision, dysuria, drowsiness, stomach discomfort

Serious side effects: glaucoma, urinary retention, hepatic dysfunction, paralytic ileus, hallucinations/delirium, QT prolongation, ventricular tachycardia

Beta 3-adrenergic receptor agonist

Main side effects: constipation, dry mouth

Serious side effects: urinary retention, hypertension

11. Statistical consideration

11.1 Sample size and rationale

Assuming a significance level of 5% bilaterally, a power of 80%, a symptom score difference between groups (OAB-q) of 10, and a standard deviation of 20, the sample size is 128 patients in both groups. Assuming a dropout rate of 15%, the target number of participants is 150 in both groups.

11.2. Analysis participants

As an intention-to-treat (ITT) analysis, all participants for whom allocation have been initiated will be included in the analysis. Only patients who adhere to this protocol (Per Protocol Set: PPS) will also be analyzed as a sensitivity analysis.

Definition of PPS: Participants who meet the following 4 criteria

(1) Patients have received all intervention treatments.

(2) Intervention interval is less than 3 weeks.

(3) Patients have not received additional medical treatment for OAB at other hospitals

(4) All measurement data are available.

11.3 Statistical analysis

11.3.1 Primary analysis

A mixed-effects model with repeated-measures (MMRM) will be used to analyze the primary outcome, the HRQoL total score of OAB-q, under the assumption of missing at random. Estimates of the least squares mean group difference, 95% confidence interval, and p-value will be calculated as the result of group × time point estimation with the change from baseline to each measurement point as the objective variable, treatment

group, time point, OAB severity, interaction between treatment group and time point as fixed effects, study participants as variable effects, and baseline values as covariates. Week 13 is the assessment point for the primary analysis. A multiple-testing procedure will be used to control for the familywise type I error rate at a two-sided significance level of 0.05. If the primary analysis at week 13 shows statistical significance, testing of other assessment points of the primary outcome is to be performed in a hierarchical manner: week 9 and week 5.

11.3.2 Secondary analyses

Secondary analyses will be performed to supplement the results of the primary analysis of the study and to further the discussion of the clinical question. The same statistical methods will be used for the secondary outcomes as for the primary outcome. For AEs, the number and rate of occurrence in each group will be calculated.

11.3.3 Subgroup analysis

The following four subgroup analyses will be performed:

1) Participants: aged 20- to 64-years-old vs 65- to 80-years-old;
2) OAB severity: moderate (OABSS: 6 to 11 points) vs severe (OABSS: 12 to 15 points);
3) Pharmacotherapy: naïve vs past vs under treatment;
4) Incontinence: no (OABSS Q4 = 0 point) vs yes (OABSS Q4 = 1 to 5 point);
5) HADS Anxiety score: normal (0 to 7 points) vs borderline or abnormal (8 to 21 points)
6) HADS Depression score: normal (0 to 7 points) vs borderline or abnormal (8 to 21 points).

When possible and/or necessary,

7) Intervention type: face-to-face vs online remote session

Age is considered the most significant risk factor for OAB, and understanding of CBT may differ depending on age, as well as the effectiveness of the intervention. We believe that the severity of OAB has a strong influence on the effectiveness of the intervention. Intervention effectiveness may differ depending on whether CBT alone or in combination with oral medication is used. Urinary incontinence is caused by weakness of the pelvic floor muscles, and the intervention effect may differ depending on the presence or absence of urinary incontinence. CBT is a psychotherapy, and treatment effects may differ in participants with anxiety or depression. Regarding the method of

intervention, the treatment effect may differ between face-to-face and online. In addition, there may be different age groups among the participants who prefer face-to-face versus online. For these reasons, subgroup analyses will be conducted for age, severity, presence of urinary incontinence, and HADS scores.

11.3.4 Sensitivity analysis

The ITT will be used as the main analysis, and the PPS will be used as the sensitivity analysis. Furthermore, sensitivity analyses included multiple imputation approach for imputing missing data will be performed, with missing at random assumptions.

12. Study Period

12.1 Participant enrollment period

Two years and 6 months from the date of approval.

12.2 Participant follow-up period

Four weeks of CBT and 8 weeks of post-treatment follow-up (total 12 weeks).

12.3 Study duration

Three years from the date of approval.

13. Additional invasions and anticipated risks and benefits of the study

There will be a time burden associated with the completion of the self-administered symptom rating scale and the implementation of the CBT program, but no other specific risks are anticipated. No AEs were observed in the pilot study. The expected benefit is that the CBT incorporates behavioral therapy, which has originally been shown to be effective, and may lead to improvement of symptoms of OAB.

14. Availability of compensation for adverse health effects

Although not covered by insurance, this treatment incorporates behavioral therapy recommended in the guidelines for the treatment of OAB and is an intervention within the scope of insurance coverage. Therefore, we believe that the anticipated health hazards can also be covered by insurance. For this reason, no compensation for health damage is provided. In the event of health damage, each individual will be examined and treated by the insurance.

15. Protection of personal information

15.1 Timing and method of anonymization of samples and information

All data will be entered within REDCap. After entry, the data will be anonymized with serial numbers. After anonymization, research participants will be identified by manipulating the numbers given to them.

15.2 Methods for managing the correspondence table

A new correspondence table will be created and stored at each facility. The correspondence list will be stored securely in a computer with a password.

15.3 Items of personal information to be retained or used, security control measures, and points to note

Only the items obtained from the questionnaire will be used. Specifically, the following information will be used: age, height, weight, medical history, childbirth experience, menopause, last education, marital history, occupational history, treatment for voiding dysfunction, primary outcome, secondary outcomes, and AEs. As a security measure, data will be managed in REDCap, access to REDCap will be restricted to researchers, and all access will be logged in the system.

15.4 Persons responsible for information management of the entire research organization

Principal Investigator: Takashi Kobayashi

15.5 Handling of data after withdrawal of consent

Data after withdrawal of consent will not be used. Data obtained before the withdrawal of consent by the person withdrawing consent will also not be used.

16. Explanation and consent

16.1 Rules and regulations to be observed

In accordance with the "Declaration of Helsinki of the World Medical Association" and the "Ethical Guidelines for Life Sciences and Medical Research Involving Human Participants," we guarantee that the research participants can discontinue this research at any time if they so desire. When a research participant requests discontinuation of participation in the research, the use of such data will be discontinued.

16.2 Written explanation and consent form

The attached explanation and consent forms will be used.

17. Methods for storage and disposal of samples and information

17.1 Period of storage of information

Registered data will be stored at the Department of Urology, Kyoto University School of Medicine, and at each institution for 10 years after publication.

17.2 Method of information storage (measures to prevent leakage, mix-up, theft, loss, etc.)

Data collected at each institution will be stored in a database on REDCap, with each institution's individual participant identifiers deleted and a new research ID assigned. Access to REDCap will be restricted to researchers to prevent leakage, mix-up, theft, or loss. In addition, the system keeps a history of all accesses, which should allow for contingency measures.

17.3 Disposal after the retention period, if any

After the retention period, if consent is obtained, the correspondence list will be destroyed and uploaded to the UMIN ICDR in a form that does not identify specific individuals and stored semi-permanently.

17.4 When providing and receiving information from other research institutions, create and maintain records related to the provision of such information

We are considering the possibility of providing information to other research periods by uploading it to the UMIN ICDR. Since the UMIN ICDR can only be used to provide information to designated researchers, the creation and management of records will be shared. Conversely, the same will apply to the provision of information.

18. Cost sharing and honoraria

The intervention group will require office visits for intervention (weeks 1, 2, 3, and 4) and follow-up (weeks 5, 9, and 13); the control group will require office visits for allocation (week 1) and follow-up (weeks 5, 9, and 13). The cost of the hospital visits (re-consultation fees only) will be borne by the study participants within the insurance reimbursement. However, if the participants wish to participate in the online intervention, the consultation fee at the time of intervention will not be required. As for the gratuity, both groups will receive a gift certificate worth 1,000 yen at the week 5, 9 and 13 visits after answering each assessment item.

19. Research funding and conflict of interest

This study is funded by a donation (Grant-in-Aid for Urology Research) and Grant-in-Aid for Young Scientists (20K18964) from the Ministry of Education, Culture, Sports, Science and Technology of Japan.
Conflicts of interest are appropriately reviewed by the Kyoto University Clinical Research Conflict of Interest Review Committee in accordance with the Kyoto University Conflict of Interest Policy and the Kyoto University Conflict of Interest Management Regulations.

20. Content and method of reporting to the Ethical Review Committee and the head of the research institution

If facts or information that undermine or may undermine the ethical validity or scientific rationality of the research are obtained, a report on safety information will be made promptly. If facts or information that undermine or may undermine the appropriateness of research implementation or the reliability of research results are obtained, a report of nonconformity will be promptly submitted. Annual reports will be made annually. Reports on discontinuation or termination will be made as appropriate. Conference presentations and papers of the results will be attached to the electronic submission system in PDF format. The final publication of the results shall also be reported.

21. Attribution of research results and publication of results

The research results obtained in this study belong to Kyoto University. The results will be published in academic conferences and papers. In addition, the results will be pre-registered, updated, and registered in the public database of UMIN (accession number: UMIN000038513).

22. Measures to be taken regarding the provision of medical care to research participants after the research is conducted

After the research is conducted, efforts will be made to ensure that research participants receive the best possible treatment using the findings from the research.

23. Recruitment of participants

The recruitment for this study will include the leaflet, information sessions for the public, information sessions for physicians, introduction of the study on the laboratory's website, internet advertisement, newspaper advertisement, poster placement, and leaflet distribution.

24. Response to consultation from research participants

Research Organization: Department of Urology, Faculty of Medicine, Kyoto University
Contact Person: Satoshi Funada
TEL: 075-751-3337
Email: sfunada@kuhp.kyoto-u.ac.jp

Hospital Contact: Kyoto University Hospital, Clinical Research Consultation Service
TEL: 075-751-4748
Email: ctsodan@kuhp.kyoto-u.ac.jp

25. Actions to be taken in the event of changes or revisions to the research protocol

When changes occur in the research, the researcher will apply to the Kyoto University School of Medicine and the Kyoto University Hospital Medical Ethics Committee for changes to the application documents, obtain institutional approval, and then continue the research using the most recent version.

26. Research organization

Principal Investigator
Professor Takashi Kobayashi, Department of Urology, Graduate School of Medicine, Kyoto University
Address: 54, Shogoin Kawahara-cho, Sakyo-ku, Kyoto, 606-8507, Japan
Telephone number: 075-751-3325
E-mail address: selecao@kuhp.kyoto-u.ac.jp

Data management contact
Satoshi Funada, Graduate School of Urology, Kyoto University Graduate School of Medicine
Address: 54, Shogoin Kawahara-cho, Sakyo-ku, Kyoto, 606-8507, Japan
Telephone number: 075-751-3337
E-mail address: sfunada@kuhp.kyoto-u.ac.jp

Person responsible for statistical analysis
Ryuji Uozumi, Lecturer, Department of Medical Statistics and Bioinformatics, Graduate School of Medicine, Kyoto University
Address: 54, Shogoin Kawahara-cho, Sakyo-ku, Kyoto, 606-8507, Japan

Telephone number: 075-751-4725
E-mail address: uozumi@kuhp.kyoto-u.ac.jp

Person in charge of statistical analysis
Kentaro Ueno, Assistant Professor, Department of Medical Statistics and Bioinformatics, Graduate School of Medicine, Kyoto University
Address: 54, Shogoin Kawahara-cho, Sakyo-ku, Kyoto, 606-8507, Japan
Telephone number: 075-751-3165
E-mail address: ueno_kentaro@kuhp.kyoto-u.ac.jp

Sub-investigator (Research Planning and Supervision)
Shusuke Akamatsu, Lecturer, Department of Urology, Graduate School of Medicine, Kyoto University
Address: 54, Shogoin Kawahara-cho, Sakyo-ku, Kyoto, 606-8507, Japan
Telephone number: 075-751-3337
E-mail address: akamatsu@kuhp.kyoto-u.ac.jp

Sub-investigator (Research Planning and Supervision)
Assistant Professor Takayuki Goto, Department of Urology, Graduate School of Medicine, Kyoto University
Address: 54, Shogoin Kawahara-cho, Sakyo-ku, Kyoto, 606-8507, Japan
Telephone number: 075-751-3337
E-mail address: goto@kuhp.kyoto-u.ac.jp

Sub-investigator (Responsible for the research site)
Kentaro Ichioka, Director, Ichioka Urology Clinic
Address: Nijo-sagaru, Higashinotoin-dori, Nakagyo-ku, Kyoto, 606-8226, Japan
Telephone number: 075-231-7227
E-mail address: ichiokakentaro1003@gmail.com

Sub-investigator (Research Site Director, Interpretation of Study Results, Preparation of Articles)
Yumiko Nakagawa, Director, Asukai Hospital, Kyoto Civil Medical Federation
Address: 89 Asukai-cho, Tanaka, Sakyo-ku, Kyoto, 606-8226, Japan
Telephone number: 075-701-6111
E-mail address: medi-wind@shinwakai-min.jp

Sub-investigator (Site Investigator, Interpretation of Study Results, Preparation of Article)
Takehiko Segawa, Chief, Department of Urology, Kyoto City Hospital
Address: 1-2 Mibu Higashi Takada-cho, Nakagyo-ku, Kyoto, 604-8845, Japan
Telephone number: 075-311-5311
E-mail address: segawa@kuhp.kyoto-u.ac.jp

Sub-investigator (Supervisors)
Nagoya City University Graduate School of Medicine Department of Psychiatry, Cognitive and Behavioral Medicine
Professor Tatsuo Akechi
Address: 1 Kawasumi, Mizuho-cho, Mizuho-ku, Nagoya 467-8601, Japan
Telephone number: 052-853-8271
E-mail address: akechi@med.nagoya-cu.ac.jp

Sub-investigator (Research Planning and Supervision)
Department of Health Promotion and Behavioral Science, Division of Social and Health Medicine, Graduate School of Medicine, Kyoto University
Professor Toshi A. Furukawa
Address: Yoshida Konoe-cho, Sakyo-ku, Kyoto, 606-8507, Japan
Telephone number: 075-753-9491
E-mail address: furukawa@kuhp.kyoto-u.ac.jp

27. References

1. Homma Y, Yoshida M, Seki N, Yokoyama O, Kakizaki H, Gotoh M, et al. Symptom assessment tool for overactive bladder syndrome-overactive bladder symptom score. Urology. 2006;68(2):318–23.
2. 本間之夫，後藤百万. Overactive bladder questionnaire（OAB-q）の日本語版の作成と言語的妥当性の検討．日排尿機能会誌. 2006;241–9.
3. Uemura S, Homma Y. Reliability and validity of King's Health Questionnaire in patients with symptoms of overactive bladder with urge incontinence in Japan. Neurourol Urodyn [Internet]. 2004;23(2):94–100. Available from: http://www.ncbi.nlm.nih.gov/pubmed/14983417
4. Yalcin I, Bump RC. Validation of two global impression questionnaires for incontinence. Am J Obstet Gynecol [Internet]. 2003 Jul;189(1):98–101. Available from:

https://linkinghub.elsevier.com/retrieve/pii/S0002937803003338
5. 池田俊也, 白岩健, 五十嵐中, 能登真, 福田敬, 齋藤信也, et al. 日本語版EQ-5D-5Lにおけるスコアリング法の開発. 2015;64(1):47–55.
6. 八田 宏之, 東 あかね, 八城 博子, 小笹 晃太郎, 林 恭平, 清田 啓介, et al. Hospital Anxiety and Depression Scale日本語版の信頼性と妥当性の検討 女性を対象とした成績. 心身医学 [Internet]. 1998;38(5):309–15. Available from: https://www.jstage.jst.go.jp/article/jjpm/38/5/38_KJ00002386587/_pdf/-char/ja
7. Abrams P, Cardozo L, Fall M, Griffiths D, Rosier P, Ulmsten U, et al. The standardisation of terminology of lower urinary tract function: report from the Standardisation Sub-committee of the International Continence Society. Neurourol Urodyn [Internet]. 2002;21(2):167–78. Available from: http://www.ncbi.nlm.nih.gov/pubmed/11857671
8. 日本排尿機能学会, 日本泌尿器科学会. 過活動膀胱診療ガイドライン[第2版]. リッチヒルメディカル, 東京. 2015;
9. Homma Y, Yamaguchi O, Hayashi K. An epidemiological survey of overactive bladder symptoms in Japan. BJU Int [Internet]. 2005 Dec;96(9):1314–8. Available from: http://doi.wiley.com/10.1111/j.1464-410X.2005.05835.x
10. Vaughan CP, Johnson TM, Ala-Lipasti M a, Cartwright R, Tammela TLJ, Taari K, et al. The prevalence of clinically meaningful overactive bladder: bother and quality of life results from the population-based FINNO study. Eur Urol [Internet]. 2011;59(4):629–36. Available from: http://www.ncbi.nlm.nih.gov/pubmed/21306820
11. 井上幸恵, 小林慎, 菅谷公男. 過活動膀胱の医療経済. 日本泌尿器科学会雑誌. 2008;99(7):713–22.
12. Sexton CC, Notte SM, Maroulis C, Dmochowski RR, Cardozo L, Subramanian D, et al. Persistence and adherence in the treatment of overactive bladder syndrome with anticholinergic therapy: A systematic review of the literature. Int J Clin Pract. 2011;65(5):567–85.
13. Richardson K, Fox C, Maidment I, Steel N, Loke YK, Arthur A, et al. Anticholinergic drugs and risk of dementia: case-control study. Bmj [Internet]. 2018;k1315. Available from: http://www.bmj.com/lookup/doi/10.1136/bmj.k1315
14. Gormley EA, Lightner DJ, Faraday M, Vasavada SP. Diagnosis and treatment of overactive bladder (non-neurogenic) in adults: AUA/SUFU guideline amendment. J Urol [Internet]. 2015;193(5):1572–80. Available from: http://dx.doi.org/10.1016/j.juro.2015.01.087
15. Benner JS, Nichol MB, Rovner ES, Jumadilova Z, Alvir J, Hussein M, et al. Patient-reported reasons for discontinuing overactive bladder medication. BJU Int [Internet]. 2010 May;105(9):1276–82. Available from: http://www.ncbi.nlm.nih.gov/pubmed/19912188
16. BECK AT. THINKING AND DEPRESSION. I. IDIOSYNCRATIC CONTENT AND

COGNITIVE DISTORTIONS. Arch Gen Psychiatry [Internet]. 1963 Oct;9:324–33. Available from: http://www.ncbi.nlm.nih.gov/pubmed/14045261

17. Eccleston C, Fisher E, Law E, Bartlett J, Palermo TM. Psychological interventions for parents of children and adolescents with chronic illness. Cochrane database Syst Rev [Internet]. 2015 Apr 15;(4):CD009660. Available from: http://www.ncbi.nlm.nih.gov/pubmed/25874881

18. Zijdenbos IL, de Wit NJ, van der Heijden GJ, Rubin G, Quartero AO. Psychological treatments for the management of irritable bowel syndrome. Cochrane database Syst Rev [Internet]. 2009 Jan 21;(1):CD006442. Available from: http://www.ncbi.nlm.nih.gov/pubmed/19160286

19. Homma Y, Yoshida M, Seki N, Yokoyama O, Kakizaki H, Gotoh M, et al. Symptom assessment tool for overactive bladder syndrome--overactive bladder symptom score. Urology [Internet]. 2006 Aug;68(2):318–23. Available from: http://www.ncbi.nlm.nih.gov/pubmed/16904444

20. Coyne KS, Matza LS, Thompson CL, Kopp ZS, Khullar V. Determining the Importance of Change in the Overactive Bladder Questionnaire. J Urol [Internet]. 2006 Aug;176(2):627–32. Available from: http://www.jurology.com/doi/10.1016/j.juro.2006.03.088
