# Supplementary material for: Multicomponent Intervention for Overactive Bladder in Women: A Randomized Clinical Trial
Source: JAMA Netw Open. 2024 Mar 13;7(3):e241784. doi: 10.1001/jamanetworkopen.2024.1784 (PMC10938174; doi:10.1001/jamanetworkopen.2024.1784)
Supplement: Supplement 2. — eTable 1. HRQL Total Scores of OAB-q at Baseline, 5, 9, and 13 Weeks (Subgroup Analysis) eTable 2. HRQL Total Scores of OAB-q at Baseline, 5, 9, and 13 Weeks (Per-Protocol Set Analysis) eTable 3. HRQL Total Scores of OAB-q at Baseline, 5, 9, and 13 Weeks (Multiple Imputation) eTable 4. Secondary Outcomes at Baseline, 5, 9, and 13 Weeks eTable 5. Secondary Outcomes at Baseline, 5, 9, and 13 Weeks (PGI) eTable 6. Secondary Outcomes at Baseline, 5, 9, and 13 Weeks (Change in Pharmacotherapy) eFigure 1. Cognitive Behavior Therapy Model for Overactive Bladder eFigure 2. Example of Exposure Technique Using a Graded Exposure Task [file jamanetwopen-e241784-s002.pdf]

Supplemental Online Content

Funada S, Luo Y, Uozumi R, et al. Multicomponent intervention for overactive bladder in women: a randomized clinical trial. *JAMA Netw Open*. 2024;7(3):e241784. doi:10.1001/jamanetworkopen.2024.1784

eTable 1. HRQL Total Scores of OAB-q at Baseline, 5, 9, and 13 Weeks (Subgroup Analysis)
eTable 2. HRQL Total Scores of OAB-q at Baseline, 5, 9, and 13 Weeks (Per-Protocol Set Analysis)
eTable 3. HRQL Total Scores of OAB-q at Baseline, 5, 9, and 13 Weeks (Multiple Imputation)
eTable 4. Secondary Outcomes at Baseline, 5, 9, and 13 Weeks
eTable 5. Secondary Outcomes at Baseline, 5, 9, and 13 Weeks (PGI)
eTable 6. Secondary Outcomes at Baseline, 5, 9, and 13 Weeks (Change in Pharmacotherapy)
eFigure 1. Cognitive Behavior Therapy Model for Overactive Bladder
eFigure 2. Example of Exposure Technique Using a Graded Exposure Task

This supplemental material has been provided by the authors to give readers additional information about their work.

© 2024 Funada S et al. *JAMA Network Open.*

eTable 1. HRQoL Total Scores of the OAB-q at Baseline, 5, 9, and 13 Weeks (Subgroup Analysis)

Outcome, time point		Multicomponent intervention (n = 39) No.	Mean (SD)	LS mean changes (95% CI)	Waiting list (n = 40) No.	Mean (SD)	LS mean changes (95% CI)	Comparison between groups: Difference in LS mean changes (95% CI)	P-value	P-value for Interaction
Age										0.71
Age < 65	Baseline	15	58.3 (15.3)	NA	15	59.8 (18.9)	NA	NA	NA	
	5 wk	14	78.9 (14.9)	20.6 (13.3 to 27.9)	14	65.8 (21.9)	7.1 (-0.2 to 14.4)	13.5 (3.1 to 23.8)	0.013	
	9 wk	13	87.4 (9.2)	27.8 (20.9 to 34.8)	13	69.8 (21.4)	12.7 (5.8 to 19.6)	15.1 (5.3 to 25.0)	0.004	
	13 wk	13	90.5 (7.3)	31.1 (24.6 to 37.5)	14	71.4 (19.7)	12.8 (6.4 to 19.1)	18.3 (9.3 to 27.3)	<.001	
Age ≥ 65	Baseline	24	62.0 (17.0)	NA	25	69.7 (14.7)	NA	NA	NA	
	5 wk	23	81.8 (11.3)	16.9 (10.6 to 23.1)	24	74.4 (17.2)	5.2 (-0.4 to 10.8)	11.7 (4.2 to 19.2)	0.003	
	9 wk	23	82.3 (10.5)	17.4 (11.2 to 23.7)	24	77.4 (16.8)	8.2 (2.6 to 13.8)	9.3 (1.7 to 16.8)	0.017	
	13 wk	21	85.8 (9.0)	19.7 (13.0 to 26.3)	24	80.3 (17.2)	11.1 (5.1 to 17.1)	8.5 (0.5 to 16.6)	0.039	
OAB severity										0.63
Moderate	Baseline	36	61.4 (15.3)	NA	35	65.9 (17.1)	NA	NA	NA	
	5 wk	34	80.7 (12.2)	24.2 (-0.1 to 48.4)	33	71.6 (19.2)	4.9 (-13.9 to 23.6)	19.3 (-11.5 to 50.1)	0.177	
	9 wk	33	84.5 (9.3)	24.2 (6.7 to 41.6)	32	75.5 (19.0)	5.8 (-7.6 to 19.3)	18.3 (-3.9 to 40.6)	0.086	
	13 wk	31	88.1 (8.6)	26.0 (9.6 to 42.5)	33	77.7 (18.9)	9.0 (-3.6 to 21.6)	17.0 (-4.0 to 38.1)	0.095	
Severe	Baseline	3	51.5 (27.4)		5	66.6 (17.4)	NA	NA	NA	
	5 wk	3	80.3 (20.6)	18.5 (14.4 to 22.6)	5	68.6 (21.2)	6.3 (2.1 to 10.5)	12.2 (6.2 to 18.1)	<.001	
	9 wk	3	80.3 (20.5)	21.7 (17.4 to 26.1)	5	69.6 (16.4)	10.7 (6.2 to 15.1)	11.1 (4.8 to 17.3)	0.001	
	13 wk	3	82.1 (8.92)	24.4 (19.8 to 29.0)	5	72.8 (16.1)	12.4 (7.8 to 16.9)	12.0 (5.5 to 18.5)	<.001	
OAB medication										0.27

© 2024 Funada S et al. *JAMA Network Open.*

No prior treatment	Baseline	14	63.2 (17.6)	NA	16	73.9 (12.2)	NA	NA	NA	
	5 wk	12	82.0 (11.7)	9.9 (1.5 to 18.4)	15	80.5 (13.8)	7.0 (0.5 to 13.5)	2.9 (-6.1 to 11.9)	0.511	
	9 wk	11	88.1 (6.0)	14.4 (6.2 to 22.6)	15	84.4 (11.3)	11.0 (4.9 to 17.0)	3.4 (-4.9 to 11.8)	0.400	
	13 wk	11	90.0 (5.2)	16.5 (8.5 to 24.5)	15	84.2 (11.6)	10.8 (4.9 to 16.6)	5.8 (-2.2 to 13.7)	0.147	
Past treatment	Baseline	9	53.4 (18.9)	NA	9	53.7 (18.1)	NA	NA	NA	
	5 wk	9	73.5 (13.7)	15.5 (4.5 to 26.6)	8	60.8 (22.0)	1.9 (-9.4 to 13.2)	13.6 (0.2 to 27.1)	0.048	
	9 wk	9	78.7 (13.5)	20.7 (10.6 to 30.8)	8	66.4 (19.3)	7.5 (-2.8 to 17.8)	13.2 (1.6 to 24.7)	0.028	
	13 wk	8	85.8 (10.2)	25.4 (14.0 to 36.9)	8	67.4 (20.6)	8.5 (-3.2 to 20.2)	16.9 (2.8 to 31.1)	0.023	
Current treatment	Baseline	16	62.4 (13.0)	NA	15	65.0 (16.7)	NA	NA	NA	
	5 wk	16	83.7 (11.9)	24.5 (15.6 to 33.4)	15	67.5 (19.4)	7.4 (-2.3 to 17.0)	17.1 (7.4 to 26.9)	0.001	
	9 wk	16	84.6 (9.7)	25.4 (16.2 to 34.6)	14	69.1 (20.8)	10.1 (0.1 to 20.1)	15.3 (4.9 to 25.7)	0.005	
	13 wk	15	86.7 (9.8)	27.0 (17.3 to 36.8)	15	75.0 (20.9)	14.8 (4.4 to 25.3)	12.2 (0.9 to 23.6)	0.036	
Incontinence										0.80
Continence	Baseline	2	40.0 (6.8)	NA	3	51.7 (30.3)	NA	NA	NA	
	5 wk	2	76.4 (1.7)	34.5 (-12.6 to 81.5)	3	62.9 (27.4)	12.4 (-25.5 to 50.3)	22.0 (-39.9 to 84.0)	0.314	
	9 wk	2	76.4 (24.3)	34.5 (-12.6 to 81.5)	3	64.5 (25.3)	14.0 (-23.9 to 51.9)	20.4 (-41.5 to 82.4)	0.344	
	13 wk	1	91.2	36.7 (-6.8 to 80.2)	3	69.1 (27.6)	18.6 (-19.4 to 56.5)	18.2 (-39.1 to 75.5)	0.413	
Incontinence	Baseline	37	61.7 (15.9)	NA	37	67.2 (15.5)	NA	NA	NA	
	5 wk	35	80.9 (13.0)	17.4 (11.9 to 22.9)	35	71.9 (18.7)	5.1 (0.0 to 10.3)	12.3 (6.2 to 18.3)	<.001	
	9 wk	34	84.6 (9.5)	20.5 (15.0 to 26.0)	34	75.6 (18.1)	9.2 (4.0 to 14.3)	11.3 (5.3 to 17.4)	<.001	
	13 wk	33	87.5 (8.7)	23.3 (17.8 to 28.8)	35	77.7 (17.9)	10.9 (5.8 to 16.1)	12.4 (6.3 to 18.5)	<.001	
HADS anxiety										0.031
Normal	Baseline	22	69.2 (13.2)	NA	32	68.3 (15.6)	NA	NA	NA	
	5 wk	21	85.6 (9.0)	19.8 (9.1 to 30.5)	30	74.9 (17.1)	-2.4 (-14.3 to 9.6)	22.2 (9.1 to 35.3)	0.002	

© 2024 Funada S et al. *JAMA Network Open.*

	9 wk	21	85.4 (8.7)	27.3 (17.2 to 37.5)	29	78.5 (16.0)	1.3 (-9.4 to 12.1)	26.0 (14.6 to 37.4)	<.001	
	13 wk	20	89.1 (7.1)	28.2 (16.8 to 39.6)	30	80.3 (15.4)	4.9 (-8.1 to 18.0)	23.2 (8.4 to 38.1)	0.004	
Borderline or Abnormal	Baseline	17	49.4 (12.7)	NA	8	57.0 (19.8)	NA	NA	NA	
	5 wk	16	74.3 (14.1)	16.0 (9.8 to 22.2)	8	57.4 (21.6)	5.5 (0.1 to 10.9)	10.5 (3.8 to 17.2)	0.003	
	9 wk	15	82.5 (12.1)	15.9 (9.7 to 22.0)	8	61.1 (22.0)	9.4 (4.1 to 14.8)	6.4 (-0.2 to 13.1)	0.058	
	13 wk	14	85.4 (10.3)	19.2 (13.0 to 25.3)	8	64.7 (24.5)	10.9 (5.7 to 16.2)	8.3 (1.7 to 14.8)	0.014	
HADS depression										0.94
Normal	Baseline	25	62.0 (15.0)	NA	29	67.0 (16.9)	NA	NA	NA	
	5 wk	23	82.5 (12.5)	12.4 (-2.0 to 26.8)	28	71.4 (19.5)	3.2 (-9.4 to 15.9)	9.2 (-2.1 to 20.5)	0.105	
	9 wk	23	84.2 (10.7)	17.5 (3.0 to 32.0)	27	74.5 (18.5)	8.0 (-4.8 to 20.7)	9.5 (-2.0 to 21.1)	0.101	
	13 wk	22	87.3 (9.2)	20.2 (5.9 to 34.5)	28	77.5 (19.2)	8.4 (-3.9 to 20.7)	11.8 (1.2 to 22.3)	0.031	
Borderline or Abnormal	Baseline	14	58.0 (18.6)	NA	11	63.3 (17.4)	NA	NA	NA	
	5 wk	14	77.6 (12.7)	19.6 (13.2 to 26.0)	10	70.6 (19.4)	5.4 (-0.5 to 11.3)	14.2 (6.9 to 21.5)	<.001	
	9 wk	13	84.1 (9.7)	21.3 (15.0 to 27.6)	10	75.4 (19.8)	9.0 (3.2 to 14.8)	12.3 (5.2 to 19.5)	0.001	
	13 wk	12	88.0 (7.8)	24.1 (17.4 to 30.8)	10	75.8 (17.2)	11.5 (5.3 to 17.6)	12.7 (4.9 to 20.5)	0.002	

Abbreviations: CI, confidence interval; HADS, hospital anxiety and depression scale; LS, least squares; OAB-q, overactive bladder questionnaire; SD, standard deviation.

© 2024 Funada S et al. *JAMA Network Open.*

eTable 2. HRQoL Total Scores of the OAB-q at Baseline, 5, 9, and 13 Weeks (Per-Protocol Set Analysis)

Outcome,	Multicomponent intervention (n = 39)			Waiting list (n = 40)			Comparison between groups	
time point	No.	Mean (SD)	LS mean changes (95% CI)	No.	Mean (SD)	LS mean changes (95% CI)	Difference in LS mean changes (95% CI)	P-value
Baseline	34	66.1 (17.0)	NA	37	60.8 (17.0)	NA	NA	NA
5 wk	34	71.0 (19.4)	18.8 (13.2 to 24.3)	37	81.6 (12.4)	5.3 (0.1 to 10.4)	13.5 (7.5 to 19.5)	<.001
9 wk	34	74.7 (18.6)	22.3 (16.9 to 27.8)	37	85.1 (9.5)	9.3 (4.2 to 14.4)	13.0 (7.1 to 18.9)	<.001
13 wk	34	76.6 (18.5)	24.8 (19.2 to 30.3)	37	87.6 (8.6)	11.5 (6.3 to 16.6)	13.3 (7.2 to 19.3)	<.001

Abbreviations: CI, confidence interval; LS, least squares; OAB-q, overactive bladder questionnaire; SD, standard deviation.

© 2024 Funada S et al. *JAMA Network Open.*

eTable 3. HRQoL Total Scores of the OAB-q at Baseline, 5, 9, and 13 Weeks (Multiple Imputation)

Outcome, time point	Multicomponent intervention (n = 39)			Waiting list (n = 40)			Comparison between groups	
	No.	Mean (SD)	LS mean changes (95% CI)	No.	Mean (SD)	LS mean changes (95% CI)	Difference in LS mean changes (95% CI)	P-value
Baseline	39	60.6 (16.2)	NA	40	66.0 (16.9)	NA	NA	NA
5 wk	37	80.7 (12.6)	18.0 (12.5 to 23.4)	38	71.2 (19.2)	5.8 (0.7 to 10.8)	12.2 (6.3 to 18.1)	<.001
9 wk	36	84.2 (10.2)	21.0 (15.5 to 26.4)	37	74.7 (18.6)	9.7 (4.6 to 14.8)	11.3 (5.4 to 17.1)	<.001
13 wk	34	87.6 (8.6)	23.6 (18.1 to 29.1)	38	77.0 (18.4)	11.7 (6.5 to 16.8)	11.9 (6.0 to 17.9)	<.001

Abbreviations: CI, confidence interval; LS, least squares; OAB-q, overactive bladder questionnaire; SD, standard deviation.

© 2024 Funada S et al. *JAMA Network Open.*

eTable 4. Secondary Outcomes at Baseline, 5, 9, and 13 Weeks

Outcome, time point	Multicomponent intervention (n = 39) No.	Mean (SD)	LS mean changes (95% CI)	Waiting list (n = 40) No.	Mean (SD)	LS mean changes (95% CI)	Comparison between groups Difference in LS mean changes (95% CI)	P-value
OABq								
Symptom bother								
Baseline	39	56.0 (21.6)	NA	40	51.6 (20.8)	NA	NA	NA
5 wk	37	33.4 (21.0)	-19.9 (-27.9 to -12.0)	38	38.7 (24.9)	-12.9 (-20.4 to -5.4)	-7.0 (-16.1 to 2.0)	0.126
9 wk	36	28.5 (16.0)	-24.5 (-31.7 to -17.2)	37	35.3 (23.3)	-16.5 (-23.2 to -9.8)	-8.0 (-15.8 to -0.2)	0.045
13 wk	34	22.4 (14.0)	-30.6 (-37.5 to -23.6)	38	32.8 (21.3)	-18.7 (-25.2 to -12.3)	-11.8 (-19.1 to -4.6)	0.002
Coping behaviors								
Baseline	39	50.8 (22.5)	NA	40	54.9 (24.0)	NA	NA	NA
5 wk	37	76.2 (19.4)	24.3 (16.4 to 32.2)	38	63.7 (27.3)	9.1 (1.6 to 16.6)	15.2 (6.5 to 23.9)	0.001
9 wk	36	82.1 (15.5)	29.4 (21.5 to 37.2)	37	67.0 (25.6)	13.2 (5.9 to 20.6)	16.1 (7.6 to 24.6)	<.001
13 wk	34	86.6 (12.5)	33.3 (25.6 to 41.0)	38	70.1 (25.2)	15.5 (8.3 to 22.6)	17.8 (9.6 to 26.0)	<.001
Concern/worry								
Baseline	39	61.4 (18.9)	NA	40	67.6 (20.0)	NA	NA	NA
5 wk	37	84.1 (14.2)	20.7 (14.3 to 27.1)	38	72.7 (21.4)	6.2 (0.3 to 12.2)	14.5 (7.5 to 21.4)	<.001
9 wk	36	87.1 (9.5)	23.2 (17.2 to 29.3)	37	77.3 (21.0)	11.1 (5.5 to 16.8)	12.1 (5.7 to 18.6)	<.001
13 wk	34	91.0 (7.4)	26.2 (20.0 to 32.5)	38	78.3 (21.9)	11.8 (6.1 to 17.6)	14.4 (7.8 to 21.1)	<.001
Sleep								
Baseline	39	54.5 (21.1)	NA	40	62.4 (19.4)	NA	NA	NA
5 wk	37	73.5 (15.4)	16.4 (9.7 to 23.2)	38	66.4 (23.5)	5.2 (-1.2 to 11.6)	11.2 (3.7 to 18.8)	0.004
9 wk	36	75.2 (14.7)	18.0 (11.6 to 24.5)	37	68.8 (20.0)	7.2 (1.2 to 13.2)	10.8 (3.9 to 17.7)	0.003

© 2024 Funada S et al. *JAMA Network Open.*

13 wk	34	78.4 (15.7)	20.4 (13.7 to 27.2)	38	73.7 (20.1)	12.5 (6.2 to 18.7)	8.0 (0.5 to 15.5)	0.037
Social interaction								
Baseline	39	81.3 (16.5)	NA	40	85.1 (15.2)	NA	NA	NA
5 wk	37	90.2 (13.0)	5.5 (1.1 to 9.9)	38	86.0 (15.4)	-0.9 (-5.0 to 3.3)	6.4 (1.4 to 11.4)	0.012
9 wk	36	92.3 (11.8)	7.5 (3.1 to 11.9)	37	89.4 (14.4)	2.9 (-1.3 to 7.0)	4.6 (-0.3 to 9.6)	0.066
13 wk	34	93.5 (9.4)	8.2 (4.0 to 12.4)	38	89.8 (13.7)	2.9 (-1.0 to 6.8)	5.3 (0.7 to 9.9)	0.025
OABSS total score								
Baseline	39	8.8 (2.0)	NA	40	8.9 (2.2)	NA	NA	NA
5 wk	37	5.5 (3.4)	-3.0 (-4.2 to -1.7)	38	6.7 (3.0)	-1.9 (-3.1 to -0.8)	-1.0 (-2.3 to 0.2)	0.100
9 wk	36	5.7 (3.1)	-2.7 (-3.9 to -1.5)	37	6.9 (2.9)	-1.8 (-2.9 to -0.7)	-0.9 (-2.1 to 0.3)	0.138
13 wk	35	4.9 (2.9)	-3.6 (-4.8 to -2.5)	38	6.0 (2.6)	-2.6 (-3.6 to -1.5)	-1.0 (-2.1 to 0)	0.059
KHQ								
General health perception								
Baseline	39	48.7 (19.0)	NA	40	40.6 (21.7)	NA	NA	NA
5 wk	37	35.1 (21.6)	-14.7 (-21.3 to -8.1)	38	38.2 (20.7)	-7.0 (-13.4 to -0.7)	-7.6 (-15.0 to -0.2)	0.044
9 wk	36	37.5 (20.3)	-11.8 (-19.1 to -4.5)	37	39.9 (21.6)	-5.5 (-12.5 to 1.5)	-6.3 (-14.9 to 2.3)	0.147
13 wk	35	31.4 (19.5)	-17.5 (-24.3 to -10.7)	38	37.5 (19.9)	-7.7 (-14.2 to -1.3)	-9.8 (-17.5 to -2.1)	0.013
Incontinence impact								
Baseline	39	57.3 (28.6)	NA	40	52.5 (29.1)	NA	NA	NA
5 wk	37	36.0 (16.4)	-22.0 (-30.6 to -13.5)	38	42.1 (28.7)	-13.2 (-21.2 to -5.2)	-8.8 (-18.3 to 0.6)	0.067
9 wk	36	28.7 (18.1)	-29.4 (-37.7 to -21.0)	37	40.5 (27.4)	-14.8 (-22.6 to -7.0)	-14.5 (-23.7 to -5.4)	0.002
13 wk	35	25.7 (16.3)	-31.8 (-40.3 to -23.2)	38	36.0 (27.3)	-19.3 (-27.3 to -11.3)	-12.4 (-21.9 to -2.9)	0.011
Role limitations								
Baseline	39	39.3 (23.4)	NA	40	32.1 (28.3)	NA	NA	NA

© 2024 Funada S et al. *JAMA Network Open.*

5 wk	37	23.0 (23.7)	-17.7 (-26.9 to -8.5)	38	25.0 (27.1)	-11.8 (-20.5 to -3.1)	-5.9 (-16.7 to 4.9)	0.281
9 wk	36	20.8 (20.1)	-19.6 (-27.5 to -11.6)	37	21.2 (22.4)	-15.9 (-23.3 to -8.5)	-3.6 (-12.2 to 4.9)	0.400
13 wk	35	14.3 (17.2)	-25.6 (-33.8 to -17.5)	38	26.8 (25.3)	-10.0 (-17.5 to -2.5)	-15.6 (-24.5 to -6.7)	0.001
Physical limitations								
Baseline	39	45.7 (28.8)	NA	40	36.7 (29.0)	NA	NA	NA
5 wk	37	23.4 (24.7)	-18.8 (-28.7 to -8.9)	38	34.2 (29.0)	-3.3 (-12.7 to 6.2)	-15.5 (-26.4 to -4.6)	0.006
9 wk	36	19.4 (20.9)	-22.4 (-32.2 to -12.5)	37	27.9 (27.2)	-10.2 (-19.5 to -0.9)	-12.1 (-22.8 to -1.4)	0.027
13 wk	35	15.7 (19.8)	-25.2 (-34.7 to -15.7)	38	27.2 (28.1)	-10.3 (-19.2 to -1.3)	-14.9 (-25.0 to -4.9)	0.004
Social limitations								
Baseline	39	19.5 (20.1)	NA	40	14.2 (19.2)	NA	NA	NA
5 wk	37	8.1 (11.6)	-10.5 (-16.7 to -4.3)	38	16.1 (24.5)	0.5 (-5.4 to 6.3)	-10.9 (-17.9 to -3.9)	0.003
9 wk	36	6.8 (14.1)	-11.9 (-17.6 to -6.1)	37	12.9 (19.3)	-3.0 (-8.4 to 2.5)	-8.9 (-15.1 to -2.6)	0.006
13 wk	35	5.1 (12.4)	-12.4 (-18.4 to -6.4)	38	11.4 (18.4)	-4.2 (-9.9 to 1.4)	-8.2 (-14.9 to -1.5)	0.017
Personal relationships			NA					
Baseline	32	5.7 (10.9)		34	5.9 (14.1)	NA	NA	NA
5 wk	31	1.6 (5.0)	-4.5 (-8.4 to -0.6)	32	6.3 (15.7)	0.1 (-3.7 to 3.9)	-4.6 (-9.8 to 0.6)	0.084
9 wk	28	0.0 (0.0)	-5.8 (-8.1 to -3.5)	29	3.4 (9.3)	-3.1 (-5.3 to -0.9)	-2.7 (-5.5 to 0.1)	0.056
13 wk	28	0.0 (0.0)	-5.8 (-8.6 to -3.1)	30	2.8 (10.8)	-3.7 (-6.3 to -1.0)	-2.2 (-5.7 to 1.3)	0.221
Emotions								
Baseline	39	55.8 (26.4)	NA	40	47.8 (27.2)	NA	NA	NA
5 wk	37	32.1 (22.6)	-22.4 (-30.8 to -14.0)	38	35.7 (27.0)	-14.5 (-22.4 to -6.6)	-7.9 (-16.8 to 1.1)	0.083
9 wk	36	26.2 (19.2)	-26.8 (-35.4 to -18.1)	37	33.3 (26.6)	-17.6 (-25.7 to -9.4)	-9.2 (-18.6 to 0.2)	0.055
13 wk	35	19.4 (15.1)	-32.0 (-40.5 to -23.4)	38	30.4 (26.9)	-19.8 (-27.8 to -11.7)	-12.2 (-21.4 to -3.0)	0.010
Sleep/energy								

© 2024 Funada S et al. *JAMA Network Open.*

Baseline	39	40.2 (25.6)	NA	40	31.3 (25.1)	NA	NA	NA
5 wk	37	16.7 (15.2)	-22.4 (-28.9 to -16.0)	38	31.1 (26.3)	-3.7 (-9.9 to 2.4)	-18.7 (-26.0 to -11.3)	<.001
9 wk	36	16.7 (15.9)	-22.6 (-29.1 to -16.2)	37	28.4 (20.7)	-6.4 (-12.5 to -0.3)	-16.2 (-23.6 to -8.8)	<.001
13 wk	35	14.3 (17.2)	-24.1 (-30.8 to -17.4)	38	26.3 (21.8)	-8.6 (-14.8 to -2.3)	-15.5 (-23.2 to -7.8)	<.001
Severity measures								
Baseline	39	47.4 (17.8)	NA	40	34.2 (15.0)	NA	NA	NA
5 wk	37	35.0 (18.5)	-8.6 (-15.1 to -2.1)	38	29.8 (17.4)	-6.1 (-11.9 to -0.4)	-2.5 (-9.8 to 4.8)	0.497
9 wk	36	33.7 (12.5)	-10.1 (-16.0 to -4.2)	37	27.6 (16.3)	-8.7 (-13.7 to -3.6)	-1.4 (-7.6 to 4.8)	0.649
13 wk	35	29.9 (16.1)	-14.2 (-20.7 to -7.8)	38	28.2 (18.6)	-7.7 (-13.4 to -2.0)	-6.5 (-13.7 to 0.7)	0.075
HADS anxiety								
Baseline	39	6.5 (3.7)	NA	40	5.4 (3.5)	NA	NA	NA
5 wk	36	4.1 (3.3)	-2.1 (-3.3 to -1.0)	38	5.3 (4.0)	-0.3 (-1.4 to 0.8)	-1.8 (-3.2 to -0.5)	0.007
9 wk	36	3.6 (2.8)	-2.5 (-3.7 to -1.3)	37	5.1 (4.1)	-0.6 (-1.8 to 0.5)	-1.9 (-3.2 to -0.5)	0.008
13 wk	35	3.6 (2.9)	-2.4 (-3.6 to -1.2)	38	4.8 (3.8)	-0.9 (-2.0 to 0.2)	-1.5 (-2.8 to -0.3)	0.017
HADS depression								
Baseline	39	6.6 (3.8)	NA	40	5.9 (3.2)	NA	NA	NA
5 wk	36	5.4 (3.7)	-0.8 (-1.8 to 0.2)	38	5.5 (3.1)	-0.2 (-1.2 to 0.8)	-0.6 (-1.7 to 0.5)	0.282
9 wk	36	5.0 (3.2)	-1.1 (-2.1 to -0.1)	37	6.1 (2.8)	0.3 (-0.7 to 1.2)	-1.4 (-2.5 to -0.3)	0.015
13 wk	35	4.9 (3.8)	-1.2 (-2.3 to -0.1)	38	6.0 (3.5)	0.3 (-0.7 to 1.4)	-1.5 (-2.8 to -0.3)	0.019
EQ-5D-5L								
Baseline	39	0.79 (0.12)	NA	40	0.84 (0.10)	NA	NA	NA
5 wk	37	0.85 (0.10)	0.05 (0.02 to 0.08)	38	0.84 (0.10)	0.01 (-0.02 to 0.05)	0.04 (0 to 0.08)	0.052
9 wk	36	0.85 (0.10)	0.05 (0.02 to 0.09)	37	0.85 (0.10)	0.02 (-0.01 to 0.05)	0.03 (-0.01 to 0.06)	0.104
13 wk	35	0.85 (0.11)	0.05 (0.01 to 0.08)	38	0.86 (0.11)	0.03 (0 to 0.06)	0.02 (-0.02 to 0.06)	0.381

© 2024 Funada S et al. *JAMA Network Open.*

FVC								
Micturition, episodes/24h								
Baseline	39	10.1 (2.4)	NA	40	10.5 (2.7)	NA	NA	NA
5 wk	37	7.4 (1.8)	-2.7 (-3.3 to -2.1)	37	10.0 (2.6)	-0.2 (-0.7 to 0.4)	-2.5 (-3.2 to -1.9)	<.001
9 wk	35	7.6 (1.9)	-2.5 (-3.1 to -1.8)	37	9.8 (2.7)	-0.4 (-1.0 to 0.3)	-2.1 (-2.8 to -1.3)	<.001
13 wk	33	7.7 (2.2)	-2.4 (-3.1 to -1.7)	35	10.0 (2.8)	-0.4 (-1.1 to 0.3)	-2 (-2.8 to -1.2)	<.001
Urgency, episodes/24h								
Baseline	39	3.6 (3.3)	NA	40	3.9 (3.3)	NA	NA	NA
5 wk	37	2.5 (2.4)	-1.8 (-2.8 to -0.7)	36	4.0 (3.6)	-0.6 (-1.6 to 0.4)	-1.2 (-2.3 to -0.1)	0.029
9 wk	36	1.8 (2.2)	-2.4 (-3.7 to -1.2)	36	3.9 (3.8)	-0.7 (-1.8 to 0.5)	-1.8 (-3.2 to -0.4)	0.013
13 wk	33	1.6 (2.3)	-2.6 (-3.7 to -1.5)	34	3.6 (3.5)	-0.8 (-1.9 to 0.2)	-1.8 (-3.0 to -0.5)	0.006
Urge incontinence, episodes/24h								
Baseline	39	1.6 (3.1)	NA	40	1.3 (1.3)	NA	NA	NA
5 wk	37	1.2 (1.4)	-0.1 (-0.7 to 0.5)	36	1.2 (1.7)	0 (-0.6 to 0.6)	-0.1 (-0.8 to 0.6)	0.831
9 wk	36	0.9 (1.0)	-0.4 (-0.9 to 0.1)	36	0.8 (1.2)	-0.4 (-0.9 to 0)	0.1 (-0.5 to 0.6)	0.815
13 wk	33	0.5 (0.9)	-0.8 (-1.3 to -0.3)	34	0.9 (1.2)	-0.4 (-0.8 to 0.1)	-0.4 (-0.9 to 0.1)	0.149

Abbreviations: CI, confidence interval; EQ-5D-5L, EuroQol five-dimensional questionnaire; FVC, frequency voiding chart; HADS, hospital anxiety and depression scale; KHQ, King's health questionnaire; LS, least squares; OAB-q, overactive bladder questionnaire; OABSS, overactive bladder symptom score; SD, standard deviation.

A mixed-effects model with repeated measures analysis is used to estimate the LS mean difference in score changes from baseline at weeks 5, 9, and 13.

© 2024 Funada S et al. *JAMA Network Open.*

eTable 5. Secondary Outcomes at Baseline, 5, 9 and 13 Weeks (PGI)

Outcome, time point	Multicomponent intervention (n = 39)	Waiting list (n = 40)	Comparison between groups
	Responder (%)	Responder (%)	Difference in Proportions (95% CI)
PGI-I*			
5 wk	25 (64)	7 (18)	47 (25 to 62)
9 wk	23 (59)	5 (13)	46 (26 to 62)
13 wk	26 (67)	7 (18)	49 (28 to 65)
PGI-S†			
Baseline	13 (33)	17 (43)	-9 (-29 to 12)
5 wk	26 (67)	19 (48)	19 (-3 to 38)
9 wk	27 (69)	17 (43)	27 (5 to 45)
13 wk	32 (82)	21 (53)	30 (9 to 47)
Treatment satisfaction‡			
5 wk	32 (82)	16 (40)	42 (21 to 58)
9 wk	33 (85)	16 (40)	45 (24 to 61)
13 wk	33 (85)	20 (50)	35 (14 to 52)

Abbreviations: PGI-I, patient global impression-improvement; PGI-S, patient global impression-severity.

* Two categories (“very much improved” or “much improved”) are classified as responders compared with all other five categories.

† Two categories (“normal” or “mild”) are classified as responders compared with all other two categories.

‡ Two categories (“very satisfied” or “satisfied”) are classified as responders compared with all other three categories.

© 2024 Funada S et al. *JAMA Network Open.*

eTable 6. Secondary Outcomes at Baseline, 5, 9, and 13 Weeks (Change in Pharmacotherapy)

Outcome, time point	Multicomponent intervention (n = 39)	Waiting list (n = 40)
Change in pharmacotherapy		
5 wk	n = 37	n = 38
No change	32 (86%)	37 (97%)
Stop treatment	4 (11%)	1 (3%)
Drug reduction	1 (3%)	0 (0%)
Start drug treatment	0 (0%)	0 (0%)
9 wk	n = 36	n = 37
No change	33 (92%)	36 (97%)
Stop treatment	2 (6%)	1 (3%)
Drug reduction	0 (0%)	0 (0%)
Start drug treatment	1 (3%)	0 (0%)
13 wk	n = 35	n = 38
No change	32 (91%)	37 (97%)
Stop treatment	3 (9%)	1 (3%)
Drug reduction	0 (0%)	0 (0%)
Start drug treatment	0 (0%)	0 (0%)

© 2024 Funada S et al. *JAMA Network Open.*

eFigure 1. Cognitive Behavior Therapy Model for Overactive Bladder

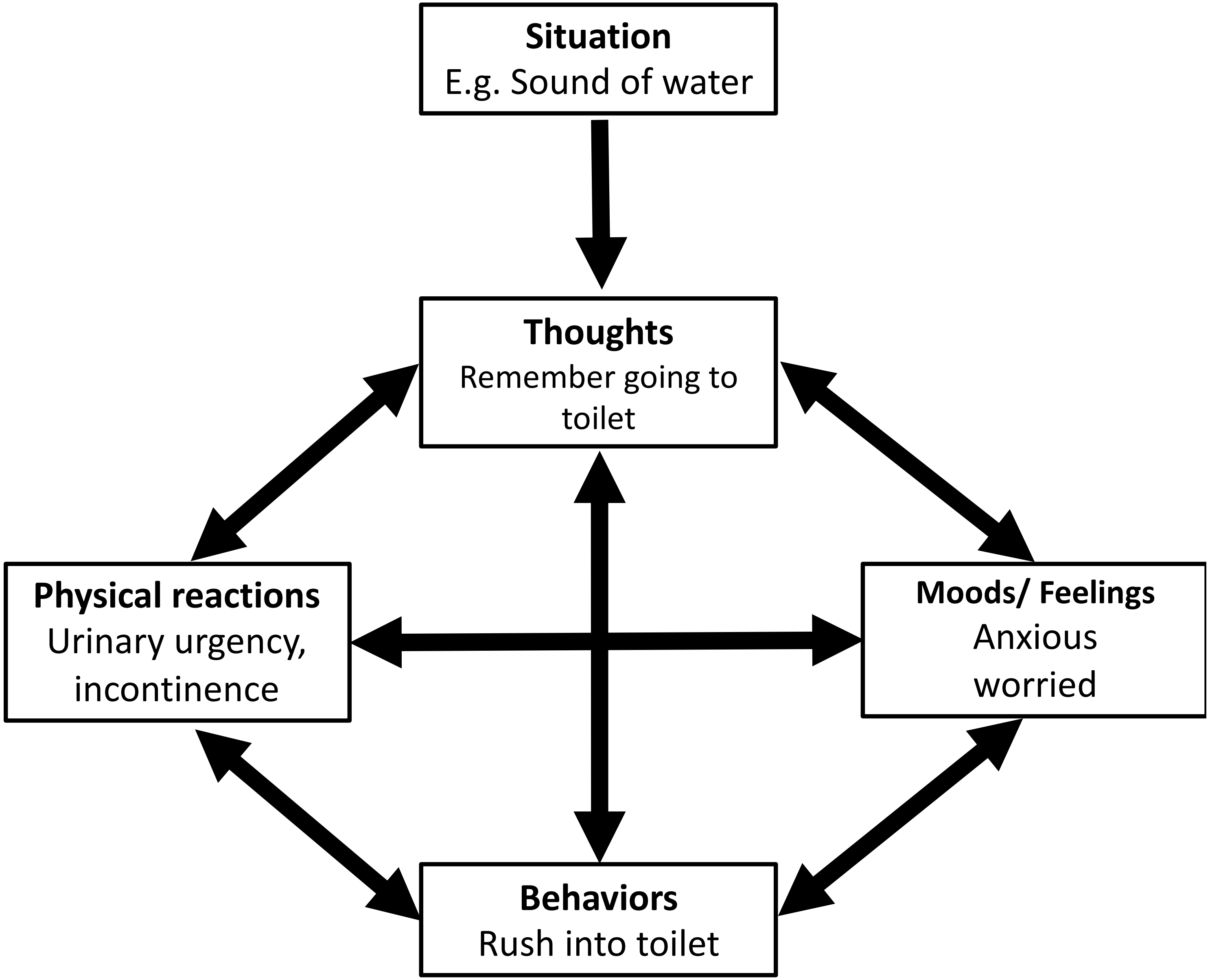

© 2024 Funada S et al. *JAMA Network Open.*

eFigure 2. Example of Exposure Technique Using a Graded Exposure Task

Exposure

Rushing to the toilet can avoid urinary urgency, but it makes a vicious circle.

Exposure to urinary urgency, which increases tolerance.

ex.)

**Goal： Trip to somewhere by bus without toilet for 3 hours**		
Step		Anxiety
1	Make urinary interval for 2 hours at home	50%
2	Make urinary interval for 3 hours at home	70%
3	Spend 3 hours outside without voiding	90%

© 2024 Funada S et al. *JAMA Network Open.*
