# Supplementary material for: Multicomponent Intervention for Overactive Bladder in Women: A Randomized Clinical Trial
Source: JAMA Netw Open. 2024 Mar 13;7(3):e241784. doi: 10.1001/jamanetworkopen.2024.1784 (PMC10938174; doi:10.1001/jamanetworkopen.2024.1784)
Supplement: Supplement 3. — Data Sharing Statement [file jamanetwopen-e241784-s003.pdf]

Data Sharing Statement

Funada. Multicomponent Intervention for Overactive Bladder in Women. *JAMA Netw Open*. Published March 13, 2024. doi:10.1001/jamanetworkopen.2024.1784

Data
Data available: Yes
Data types: Deidentified participant data
How to access data: https://center6.umin.ac.jp/cgi-bin/icdr/ctr_menu_form_reg.cgi?recptno=R000043887
When available: With publication

Supporting Documents
Document types: None

Additional Information
Who can access the data: Researchers whose proposed use of the data has been approved.
Types of analyses: Any purpose or for a specified purpose.
Mechanisms of data availability: After approval of a proposal.
